# DNA Polymerase alpha is essential for intracellular amplification of hepatitis B virus covalently closed circular DNA

**DOI:** 10.1371/journal.ppat.1007742

**Published:** 2019-04-26

**Authors:** Liudi Tang, Muhammad Sheraz, Michael McGrane, Jinhong Chang, Ju-Tao Guo

**Affiliations:** 1 Microbiology and Immunology Graduate Program, Drexel University College of Medicine, Philadelphia, PA, United States of America; 2 FlowMetric Diagnostics, Doylestown, PA, United States of America; 3 Baruch S. Blumberg Institute, Doylestown, PA, United States of America; University of California, San Diego, UNITED STATES

## Abstract

Persistent hepatitis B virus (HBV) infection relies on the establishment and maintenance of covalently closed circular (ccc) DNA, a 3.2 kb episome that serves as a viral transcription template, in the nucleus of an infected hepatocyte. Although evidence suggests that cccDNA is the repair product of nucleocapsid associated relaxed circular (rc) DNA, the cellular DNA polymerases involving in repairing the discontinuity in both strands of rcDNA as well as the underlying mechanism remain to be fully understood. Taking a chemical genetics approach, we found that DNA polymerase alpha (Pol α) is essential for cccDNA intracellular amplification, a genome recycling pathway that maintains a stable cccDNA pool in infected hepatocytes. Specifically, inhibition of Pol α by small molecule inhibitors aphidicolin or CD437 as well as silencing of Pol α expression by siRNA led to suppression of cccDNA amplification in human hepatoma cells. CRISPR-Cas9 knock-in of a CD437-resistant mutation into Pol α genes completely abolished the effect of CD437 on cccDNA formation, indicating that CD437 directly targets Pol α to disrupt cccDNA biosynthesis. Mechanistically, Pol α is recruited to HBV rcDNA and required for the generation of minus strand covalently closed circular rcDNA, suggesting that Pol α is involved in the repair of the minus strand DNA nick in cccDNA synthesis. Our study thus reveals that the distinct host DNA polymerases are hijacked by HBV to support the biosynthesis of cccDNA from intracellular amplification pathway compared to that from *de novo* viral infection, which requires Pol κ and Pol λ.

## Author summary

CCC DNA is the most refractory HBV replication intermediate under long-term antiviral therapies and is responsible for the viral rebound after treatment cessation. Therefore, understanding the biosynthesis and maintenance of cccDNA minichromosome is crucial for the development of novel antiviral therapeutics to cure chronic HBV infection. Although it has been clearly demonstrated that cccDNA biosynthesis relies on host cellular DNA repair machinery, the molecular pathways that convert rcDNA into cccDNA remain to be identified. Here we report that DNA polymerase alpha (Pol α) as well as Pol δ and ɛ are required for converting rcDNA into cccDNA through intracellular cccDNA amplification. This finding adds novel molecular insights on cccDNA biosynthesis. Further understanding the mechanism of cccDNA synthesis should reveal molecular targets for developing therapeutic agents to eradicate cccDNA and cure chronic hepatitis B.

## Introduction

Hepatitis B virus (HBV) chronically infects 257 million people worldwide [1]. Chronic HBV carriers have a higher risk of developing cirrhosis and hepatocellular carcinoma (HCC), which accounts for approximately 686,000 annual deaths [1]. Current therapies with viral polymerase inhibitors and pegylated alpha-interferon (IFN-α) can drastically reduce virus load and prevent disease progression but fail to cure the viral infection in the vast majority of treated patients [2, 3]. The reason for the failure of cure is primarily due to the inability to eradicate HBV covalently closed circular (ccc) DNA [2, 4]. The cccDNA exists in the nucleus of infected hepatocytes as a minichromosome and functions to transcribe viral RNAs and support viral replication [5, 6]. As a result, the persistence of functional cccDNA is responsible for viral rebound after the cessation of antiviral treatment [7, 8]. Therefore, understanding the mechanisms underlying cccDNA biosynthesis, maintenance and transcription regulation is essential for the development of novel antiviral therapeutics to cure chronic hepatitis B [9–11]. Unlike chromosomal DNA, cccDNA lacks a replication origin, and thereby cannot replicate through semi-conservative replication. Instead, all cccDNA molecules are converted from relaxed circular (rc) DNA in the nucleocapsids of infecting virions or mature cytoplasmic progeny nucleocapsids [12–14]. The biosynthesis of cccDNA from these two routes is designated as *de novo* synthesis and intracellular amplification, respectively. The rcDNA is a nicked double-stranded DNA with cohesive ends at both strands. The minus strand of rcDNA, synthesized from reverse transcription of pregenomic (pg) RNA, has a viral DNA polymerase covalently attached to the 5’ end and a short redundant sequence at both termini, whereas the plus strand has an 18 nt RNA primer linked to its 5’ end and is variable in length at the 3’ end. Given the unique structure of rcDNA, the conversion of rcDNA to cccDNA requires at least four steps: (*i*) the completion of plus strand DNA synthesis by DNA polymerases; (*ii*) removal of the viral polymerase covalently attached at the 5’ end of minus strand DNA and the capped RNA primer at the 5’ end of plus strand DNA *via* unknown mechanisms; (*iii*) processing of the ends of both strands of rcDNA by cellular nucleases; and (*iv*) filling in the gaps by host DNA polymerases and ligation of DNA ends by ligases [15]. These biochemical reactions have been speculated to be carried out by host cellular DNA repair machinery. Indeed, tyrosyl-DNA phosphodiesterase 2 (TDP2) [16], flap endonuclease 1 (FEN1) [17] and DNA ligases 1, 3 and 4 [18] had been shown to play essential roles in cccDNA synthesis. As for the gap-filling of rcDNA plus strand, viral polymerase activity is dispensable and an unidentified cellular DNA polymerase must have been hijacked for this process [19]. Recently, a genetic study in HepG2 cells expressing sodium-taurocholate cotransporting polypeptide (NTCP), but deficient in expression of individual cellular DNA polymerases revealed that DNA Pol κ and Pol λ were required for cccDNA formation in *de novo* HBV infection [20]. However, this work did not address the role of these cellular DNA polymerases in cccDNA biosynthesis from the intracellular amplification pathway which is crucial for maintaining a proper size of cccDNA pool in infected hepatocytes [21, 22]. Of note, our recent work showed that treatment with HBV core protein allosteric modulators (CpAMs) to induce premature uncoating of viral nucleocapsids led to reduced cccDNA formation in *de novo* infection, but increased cccDNA synthesis through intracellular amplification pathway [23]. This finding suggests that cccDNA *de novo* synthesis and intracellular amplification are differentially regulated by viral and/or host cellular factors and may recruit different DNA repair complexes to convert rcDNA into cccDNA. Accordingly, we investigated whether cccDNA intracellular amplification pathway utilizes the same DNA polymerases as cccDNA *de novo* synthesis does through a chemical genetics approach. To our surprise, we found that a non-canonical DNA repair polymerase Pol α, as well as Pol δ and ɛ, but not Pol κ and Pol λ, substantially contributed to the conversion of rcDNA to cccDNA during intracellular amplification of cccDNA in human hepatoma cells. Particularly, both inhibition of Pol α by specific inhibitors and silencing of Pol α expression by siRNA resulted in reduction of cccDNA synthesis. Conversely, inhibition of cccDNA formation by a Pol α inhibitor could be rescued by a single amino acid substitution in Pol α that abrogates the binding of the inhibitor. We also obtained evidence suggesting that Pol α is recruited to HBV rcDNA and participates in the repair of the nick in minus strand DNA. Taken together, we have identified Pol α as well as Pol δ and ɛ as novel host factors essential for cccDNA intracellular amplification and provided further evidence supporting the notion that *de novo* cccDNA synthesis and intracellular amplification of cccDNA are differentially regulated.

## Results

### Aphidicolin (APH) sensitive DNA polymerases are required for HBV cccDNA intracellular amplification

**Fig 1:**
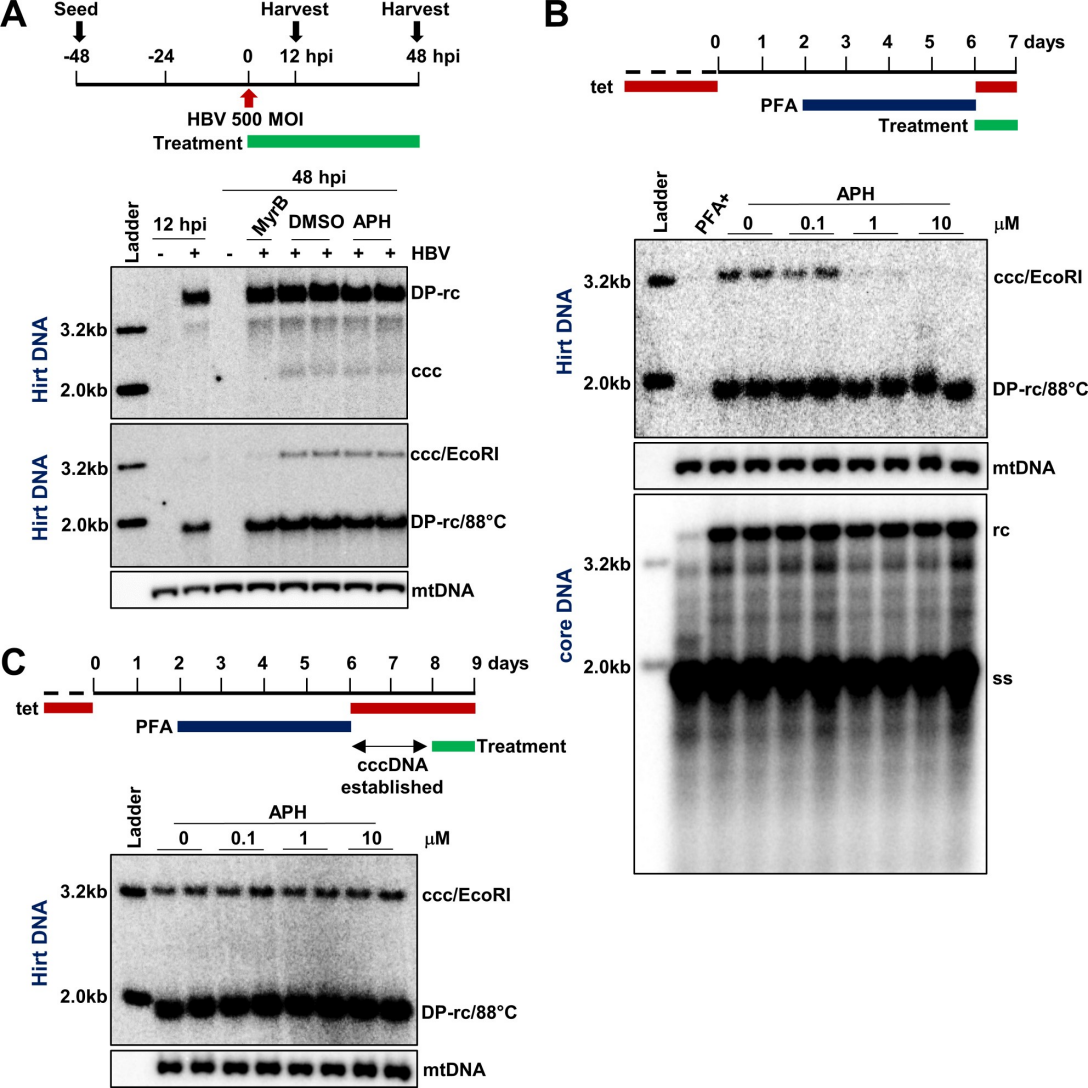
APH inhibits HBV cccDNA intracellular amplification, but not cccDNA synthesis following *de novo* infection. (A) C3A^hNTCP^ cells were inoculated with HBV at a MOI of 500 genome equivalents and treated with DMSO, 1 μM of Myrcludex B (MyrB) or 1 μM of APH starting at the time of infection. The cells were harvested at 12 h or 48 h post infection. Hirt DNA was extracted and HBV DNA was detected by Southern blot assay in its native form (upper panel) or after heat-denaturation at 88°C for 5 min to convert DP-rcDNA into single-strand DNA and followed by EcoRI digestion to linearize cccDNA to unit-length double-stranded liner DNA (lower panel). Mitochondrial (mt) DNA serves as a loading control of Hirt DNA. Each of these DNA species is denoted on the right. (B) HepAD38 cells were cultured in the presence of 2 mM PFA to arrest HBV replication from day 2 to day 6 after tet removal. On day 6, cccDNA synthesis was either inhibited by continued PFA treatment or resumed by removing PFA for 24 h. Treatment with the indicated concentrations of APH started at the removal of PFA for 24 h. Cytoplasmic HBV core DNA and protein-free (Hirt) DNA were extracted and resolved by Southern blot assays, with mt DNA as a loading control of Hirt DNA. (C) HBV cccDNA pool was allowed to be established for 48 h after removing PFA on day 6. Cells were then treated with indicated concentrations of APH for another 24 h. Hirt DNA was extracted and resolved by agarose gel electrophoresis. HBV DNA was detected by Southern blot assay, with mtDNA as a loading control.

It was reported recently that APH, an inhibitor of B family DNA polymerases that include Pol α, Pol δ and Pol ε, did not inhibit HBV cccDNA biosynthesis during *de novo* infection [20, 24]. Consistent with this report, we demonstrated that, while cccDNA synthesis in HBV infection of C3A^hNTCP^ cells can be inhibited by HBV entry inhibitor Myrcludex-B as compared to dimethyl sulfoxide (DMSO)-treated controls [25], APH treatment during the infection had no apparent influence on the levels of cccDNA (Fig 1A, upper panel). Meanwhile, we also validated the authenticity of the DNA species present in the Hirt DNA preparations, as 88°C heat denaturalization followed by EcoRI digestion converted DP-rcDNA and cccDNA into 1.6 kb single-stranded DNA and unit-length (3.2 kb) double-stranded linear (dsl) DNA, respectively (Fig 1A, lower panel) [26, 27]. Because EcoRI linearization of cccDNA after heat denaturalization of Hirt DNA increased Southern blot hybridization signals and resulted in a more accurate quantification of cccDNA, we used this cccDNA validation method as our routine cccDNA assay in this study.

As stated above, cccDNA synthesis occurs not only during *de novo* infection, but also through a process called intracellular amplification in which rcDNA in cytoplasmic progeny nucleocapsids are shuttled back into the nuclei to convert into cccDNA [12–14]. To study cccDNA intracellular amplification pathway, we utilized HepAD38 cells which support tetracycline (tet)-off inducible HBV replication and cccDNA intracellular amplification but not *de novo* infection [28]. Meanwhile, a reversible HBV polymerase inhibitor named foscarnet, or phosphonoformic acid (PFA), was applied to cell culture to arrest and synchronize HBV DNA replication primarily at full-length minus-strand DNA stage (S1 Fig). Upon release of PFA arresting, HBV DNA synthesis resumed, resulting in the sequential generation of rcDNA and cccDNA in a time-dependent manner [29]. As shown in S1 Fig, cccDNA became easily detectable after 16 h of PFA removal by Southern blot hybridization. Interestingly, distinct from its effect on cccDNA synthesis in *de novo* HBV infection, APH treatment reduced the level of cccDNA in HepAD38 cells in a dose-dependent manner, while other replication intermediates, including ssDNA, rcDNA and DP-rcDNA, were not affected (Fig 1B). Similar effect of APH on cccDNA synthesis was also observed in another HepG2-derived stable cell line (HepDES19) supporting HBV replication (S2. Fig). To investigate whether the observed reduction of cccDNA in APH-treated HepAD38 cells was due to reduced synthesis or accelerated decay, we determined the effect of APH on the established cccDNA pool. Specifically, HepAD38 cells were cultured for 48 h after release of PFA arresting to allow the establishment of cccDNA pool and then followed by treatment with indicated concentrations of APH for 24 h. As shown in Fig 1C, APH treatment did not apparently alter the level of established cccDNA. This result thus implies that APH specifically reduced cccDNA synthesis rather than accelerated its decay. Moreover, the effect of APH on cccDNA synthesis is independent of PFA arresting of HBV DNA synthesis, because in the absence of PFA arresting, treatment of HepAD38 cells with APH from day 6 to day 8 after tet removal similarly reduced the level of cccDNA (S3 Fig). Collectively, these results indicate that one or multiple APH sensitive DNA polymerases might be required for HBV cccDNA synthesis *via* intracellular amplification pathway, but not *de novo* infection of hepatocytes.

### Involvement of different DNA polymerases in *de novo* cccDNA synthesis versus intracellular cccDNA amplification

**Fig 2:**
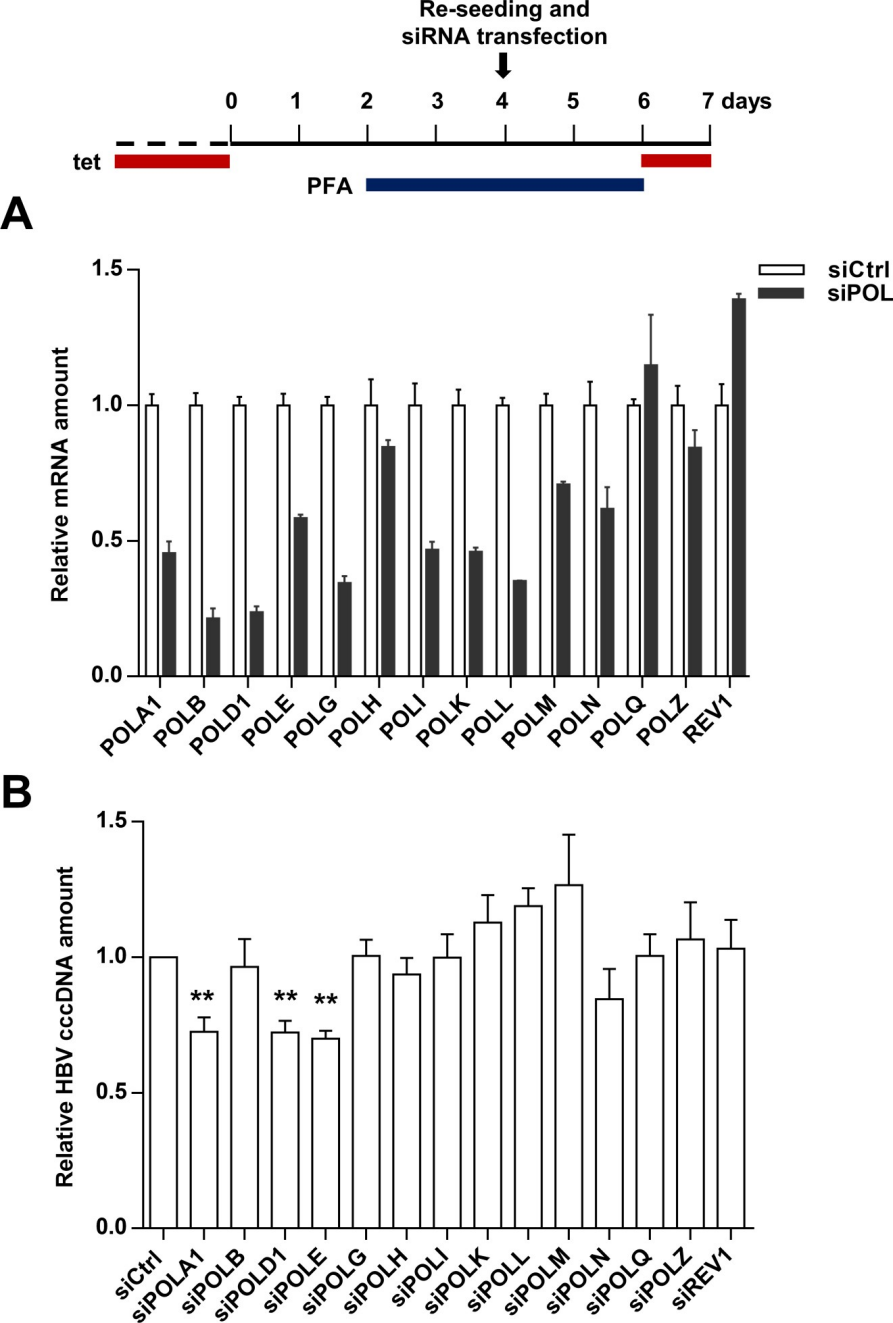
siRNA screening of host cellular DNA polymerases required for intracellular cccDNA amplification. HepAD38 cells were cultured in the presence of 2 mM PFA from day 2 to day 6 after tet removal. At day 4, the cells were re-seeded and transfected with 10 pmol siRNA targeting different cellular DNA polymerases by using RNAiMAX. (A) Total RNA was extracted at 48 h after transfection. mRNA of each indicated DNA polymerase was quantified by RT-qPCR and presented as relative amount in comparison with that in cells transfected with scramble siRNA (mean ± SD; n = 3). (B) From 48 h to 72 h after siRNA transfection (day 6 to day 7 after tet removal), cccDNA synthesis was resumed by removal of PFA from culture medium. Hirt DNA was extracted and HBV DNA was detected by Southern blot assay. The intensity of HBV cccDNA band was measured by ImageJ and presented as relative amount in comparison with that in cells transfected with scramble siRNA. Data represent 3 independent experiments (mean ± SD). Data were analyzed by two-tailed Student’s t-test (unpaired), ** *P* < 0.01.

The distinct effects of APH on HBV cccDNA biosynthesis imply that different host DNA polymerases are hijacked by the two pathways to repair rcDNA into cccDNA. Knowing Pol κ and Pol λ contribute to *de novo* cccDNA synthesis [20], we performed a focused siRNA screen to identify which cellular DNA polymerases are required for intracellular cccDNA amplification in HepAD38 cells. Using a set of siRNAs that were previously used in *de novo* cccDNA synthesis screening [20, 30], we achieved efficient knockdown for the majority of the cellular DNA polymerases in HepAD38 cells (Fig 2A) and demonstrated that knocking down the expression of only Pol α, Pol δ or Pol ε resulted in significant reduction of cccDNA amplification (Fig 2B). These results are consistent with the results that APH, an inhibitor of the B family DNA polymerases, specifically inhibits cccDNA amplification, but not *de novo* cccDNA synthesis (Fig 1B). Intriguingly, knocking down the expression of polymerases Pol κ or Pol λ, which had been shown to be essential for *de novo* cccDNA synthesis [20], did not apparently affect cccDNA intracellular amplification (Fig 2B). Of note, due to the insufficient knockdown of POL H, Q, Z and REV1 expression (Fig 2A), their roles in cccDNA amplification cannot be determined. Nevertheless, these results further strengthen the notion that different host cellular DNA polymerases preferentially involve in cccDNA synthesis from *de novo* infection and intracellular amplification pathways.

### Pol δ may play a role in cccDNA amplification

Amplification of cccDNA was inhibited by both APH treatment (Fig 1B) and silencing of three B family DNA polymerases (Fig 2B). These results suggest all the three DNA polymerases play a role in the conversion of rcDNA to cccDNA, presumably by completing plus strand DNA synthesis and/or catalyzing DNA strand elongation during DNA end processing. In fact, both Pol δ and ɛ are best known as a replicative DNA polymerase that catalyzes both lagging and leading strand DNA synthesis during semi-conservative DNA replication [31], they also play an important role in long-patch base excision repair and nucleotide excision repair [31]. We thus postulated that these two DNA polymerases may primarily mediate the inhibitory effect of APH on cccDNA synthesis. To investigate this hypothesis, Pol δ1 expression in HepAD38 cells was knocked out by CRISPR-Cas9 to generate a cell line HepAD38-*POLD1*^-/-^. The loss of Pol δ1 expression was confirmed by a Western blot assay (S4 Fig, panel A). Sanger sequencing of the gRNA targeting genomic DNA region further confirmed the disruption and indel mutation of two *POLD1* gene alleles (S4 Fig, panel B). In agreement with our siRNA screening results (Fig 2B), the deficiency of Pol δ1 hampered cccDNA amplification (S4 Fig, panel C). Ironically, APH treatment of HepAD38-*POLD1*^-/-^ cells still significantly reduced cccDNA amplification (S4 Fig, panel C), suggesting that another APH sensitive DNA polymerase is also required for cccDNA amplification and mediates APH suppression of cccDNA synthesis. Moreover, overexpression of Pol δ1 in HepAD38 cells as well as partial reconstitution of Pol δ1 expression in HepAD38-*POLD1*^-/-^ cells by plasmid transfection not only increased the level of cccDNA, but also proportionally increased the level of DP-rcDNA (S4 Fig, panel D). Because APH specifically reduced cccDNA but not DP-rcDNA (S4 Fig, panel C), these results further argue a critical role of other APH-sensitive polymerases in cccDNA amplification.

### Pol α is required for cccDNA intracellular amplification

**Fig 3:**
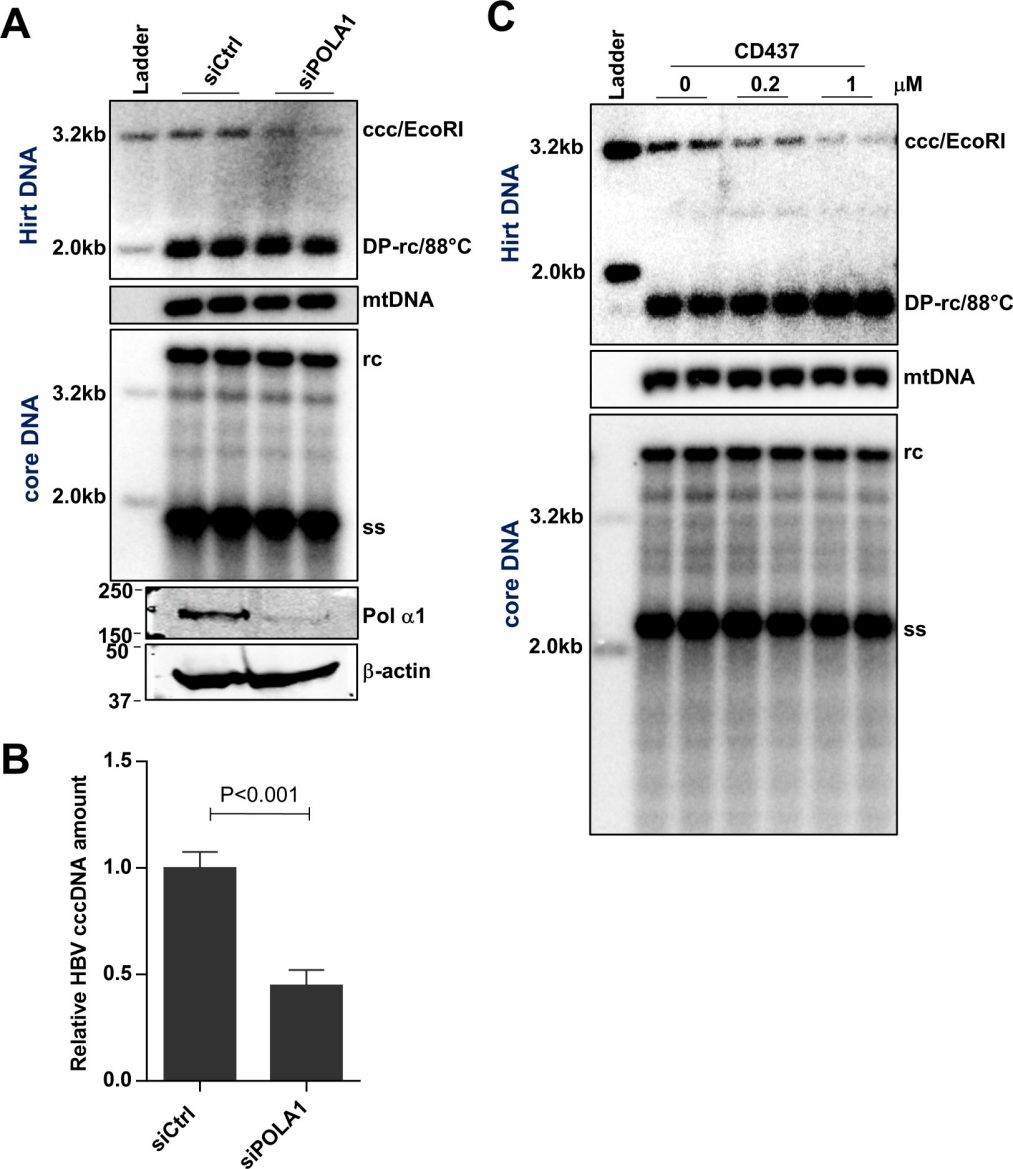
Knocking down Pol α expression or inhibition of its polymerase activity reduces HBV cccDNA synthesis. (A) HepAD38 cells were cultured in the presence of 2 mM PFA from day 2 to day 6 after tet removal. At day 4, the cells were re-seeded and transfected with 10 pmol siRNA targeting POLΑ1 or scramble siRNA, by using RNAiMAX. From 48 h to 72 h after siRNA transfection (day 6 to day 7 after tet removal), PFA was removed to allow cccDNA synthesis. The cells were harvested at 72 h after siRNA transfection, and protein levels of Pol α1 and β-actin were determined by Western blot assays. Cytoplasmic HBV core DNA and Hirt DNA were extracted and detected by Southern blot assays, with mtDNA as a loading control of Hirt DNA. (B) The intensity of HBV cccDNA band was quantified by ImageJ and presented as relative amount in comparison with that in cells transfected with scramble siRNA. Data represent 6 independent experiments (mean ± SD). Data were analyzed by two-tailed Student’s t-test (unpaired). (C) HepAD38 cells were cultured in the presence of 2 mM PFA to arrest HBV replication from day 2 to day 6 after tet removal. On day 6, cccDNA synthesis was allowed by removing PFA for 24 h and treated with the indicated concentrations of CD437. Cytoplasmic HBV core DNA and Hirt DNA were extracted and detected by Southern blot assays, with mtDNA as a loading control of Hirt DNA.

Pol α1, another APH-sensitive DNA polymerase, was also identified by siRNA screening to be required for cccDNA amplification in HepAD38 cells (Fig 2). Pol α1 is a component of the primosome complex. Once primase has created an RNA primer, Pol α starts DNA synthesis to elongate the primer at replication origins and on Okazaki fragments by approximately 20 nucleotides, from which the other replicative DNA polymerases, Pol δ or Pol ε, catalyze further elongation of the DNA chain [31, 32]. To investigate the role of Pol α in cccDNA amplification, we first confirmed our siRNA screening result by using additional Pol α1 siRNAs to deplete its expression and demonstrated that silencing of Pol α1 specifically resulted in cccDNA reduction compared with that in cells transfected by scramble siRNA (Fig 3A and 3B). Although APH treatment could further reduce the level of cccDNA in cells that Pol α1 expression was knocked down by siRNA (S5 Fig), the results clearly indicate that Pol α plays an important role in cccDNA amplification and other members of group B family DNA polymerases also contribute to cccDNA amplification. Pol α1 is the catalytic subunit of primosome which also contains a regulatory subunit Pol α2 and two primase subunits PRIM1 and PRIM2. In addition to Pol α1, knockdown of the regulatory subunit Pol α2 also reduced cccDNA amplification (S6 Fig), suggesting Pol α1 most likely couples with Pol α2 in regulating cccDNA amplification. Unfortunately, our repeated attempts to knock down each of the two primase subunits were not successful.

**Fig 4:**
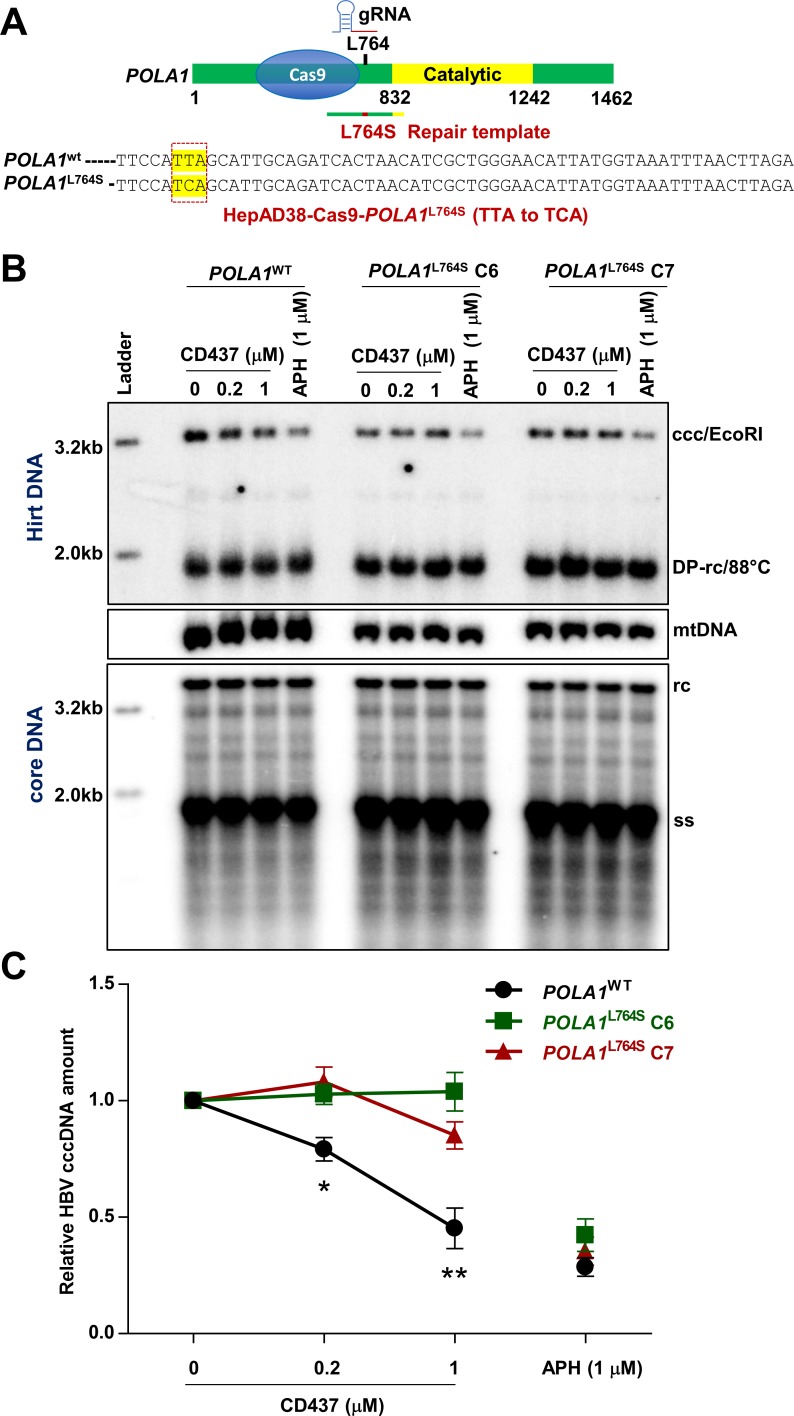
Single amino acid substitution of Pol α abolishes CD437 inhibition of cccDNA synthesis. (A) Schematic representation of CRISPR Knock-in strategy to generate HepAD38-Cas9-*POLΑ1*^L764S^ cells. The DNA sequencing results are highlighted to indicate the successful editing of TTA to TCA which results in L764S mutation. (B) HepAD38-Cas9-*POLΑ1*^WT^ and two independent clones of HepAD38-*POLΑ1*^L764S^ C6 and C7 were cultured in the presence of 2 mM PFA from day 2 to day 6 after tet removal. On day 6, cccDNA synthesis was resumed by removing PFA and the cells were treated with the indicated concentrations of CD437 or APH started at the removal of PFA for 24 h. Cytoplasmic HBV core DNA and Hirt DNA were extracted and detected by Southern blot assays, with mtDNA as a loading control of Hirt DNA. (C) The intensity of HBV cccDNA band was quantified by ImageJ and presented as relative amount in comparison with that in mock-treated correspondent cell. Data represent 4 independent experiments (mean ± SEM). Data were analyzed by two-tailed Student’s t-test (unpaired), *P < 0.05, **P < 0.01.

CD437, a recently identified Pol α1 specific inhibitor [33], dose dependently reduced cccDNA levels in HepAD38 and HepDES19 cells, while the amount of rcDNA was not significantly affected (Fig 3C and S2 Fig). As expected, cccDNA reduction was due to impaired cccDNA formation, rather than accelerated cccDNA decay because the stability of existing cccDNA was not affected by CD437 treatment (S7 Fig). It had been reported that L764 is a key residue for Pol α1 to interact with CD437 and L764S mutation renders Pol α1 resistance to CD437 [33]. Importantly, this residue is outside of the Pol α1 catalytic domain, thereby inferring that the mutation does not affect Pol α1 function [33]. To validate that CD437 suppresses cccDNA amplification via specific targeting of Pol α1, we utilized a CRISPR knock-in strategy to edit Pol α1 into Pol α1^L764S^ in HepAD38 cells. The principle of CRISPR knock-in design is depicted in Fig 4A. The successful gene editing of TTA to TCA was confirmed by Sanger sequencing of genomic DNA regions spanning the *POLΑ1* L764 position for acquired individual clones (Fig 4A). In all tested HepAD38-Pol α1^L764S^ clones, cccDNA amplification was resistant to CD437 treatment as comparing to parental cells expressing wild-type Pol α1 (*POLΑ1*^WT^) (S8 Fig). This phenomenon was further validated with two representative clones harboring the *POLΑ1* L764S mutation (*POLΑ1*^L764S^ C6 and C7). As shown in Fig 4B and 4C, although 0.2 μM and 1 μM of CD437 treatment significantly reduced cccDNA amplification in *POLΑ1*^WT^ cells, it did not affect cccDNA amplification in *POLΑ1*^L764S^ C6 and C7 cells. As a control, cccDNA amplification in all these cells was still sensitive to APH treatment (Fig 4B and 4C). These results thus indicate that CD437 inhibits cccDNA amplification by specifically targeting Pol α1 and suggest that DNA Pol α is indeed involved in cccDNA amplification in HepAD38 cells.

### The effect of Pol α in cccDNA formation is direct and rapid

**Fig 5:**
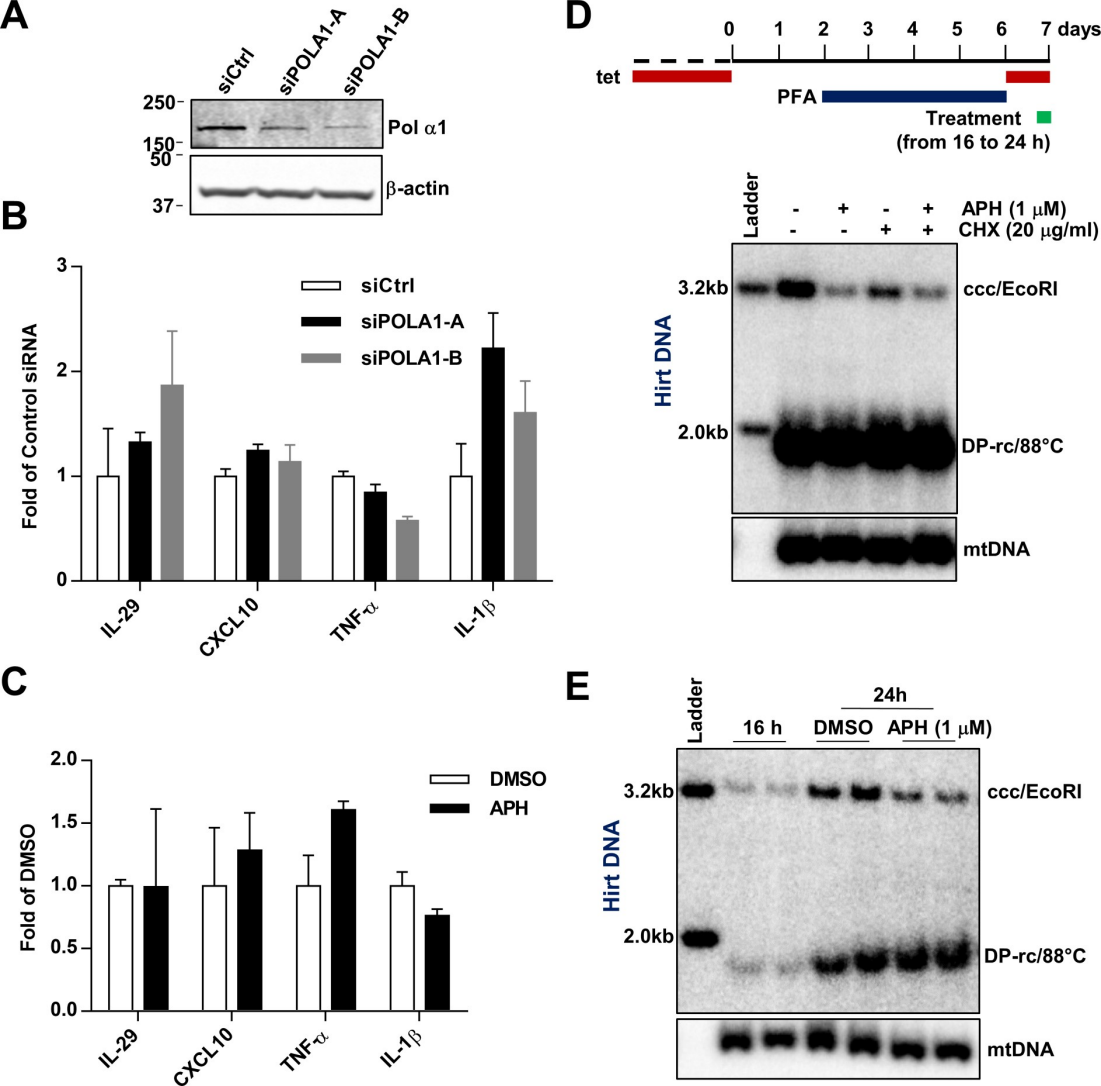
The effect of APH and CD437 on cccDNA formation is rapid and direct. (A) Seventy-two hours after transient transfection of indicated siRNAs in HepAD38 cells, protein levels of Pol α1 and β-actin were determined by Western blot assays. (B) The mRNA levels of IL-29, CXCL10, TNF-α and IL-1β were measured by qRT-PCR and presented as fold over that in cells transfected by control siRNA (mean ± SD; n = 3). (C) HepAD38 cells were mock-treated (DMSO) or treated with 1 μM APH for 8 h. Expression of the indicated cytokine mRNA and β-actin mRNA were measured by qRT-PCR and were presented as fold over that in DMSO treated cells (mean ± SD; n = 3). (D and E) HepAD38 were cultured in the presence of 2 mM PFA from day 2 to day 6 after tet removal. From day 6, cccDNA synthesis was initiated by removing PFA for 24 h, with indicated compound treatments during the last 8 h (16 to 24 h) of cccDNA synthesis. Hirt DNA was extracted and resolved by Southern blot hybridization, with mtDNA as a loading control.

In addition to its role in DNA replication, Pol α also localizes in the cytoplasm and is responsible for the generation of cytosolic DNA:RNA hybrids to antagonize pattern recognition receptor cyclic GMP-AMP synthase (cGAS), which prevents spontaneous activation of type-I interferon response [34]. To determine whether suppression of Pol α expression or its enzymatic activity in HepAD38 cells induces interferon and proinflammatory response which subsequently activates antiviral protein expression and hampers cccDNA amplification, we examined the expression of selected key cytokines (IL-29, CXCL10, TNF-α and IL-1β) upon silencing of Pol α1 by two different siRNAs or inhibition of Pol α activity by APH. No induction of these cytokines had been observed under these treatment conditions (Fig 5A–5C). In agreement with these results, the inhibitory effect of APH on cccDNA amplification did not rely on new protein synthesis, as cccDNA amplification could be efficiently inhibited by APH under the condition that protein synthesis was blocked by cycloheximide (Fig 5D). Moreover, inhibition of Pol α by APH for as short as 8 h was sufficient to inhibit cccDNA amplification (Fig 5E).

Because inhibition of group B DNA polymerases can inhibit cellular DNA synthesis and APH is commonly used for arresting cell cycle at S phase [35]. Therefore, it is possible that the observed reduction of cccDNA synthesis by inhibition of Pol α and other group B DNA polymerases was due to disruption of cell cycle progression of HepAD38 cells. Although our cccDNA synthesis assays were performed in confluent cultures, we would like to experimentally determine whether APH and CD437 treatment altered cell cycle progression under this condition. As shown in S9 Fig, flow cytometry analyses demonstrated that treatment of HepAD38 cells cultured in the sub-confluent condition with APH or CD437 arrested the cells in S phase, whereas culturing HepAD38 cells under the confluent condition which our cccDNA synthesis assays were performed arrested the cells in G1/S or G2/S phases and APH or CD437 treatment for 24 h did not change the cell cycle distribution. Furthermore, Western blot analysis of histone H3 Ser10 phosphorylation, a marker of mitosis [36], confirmed that the cells in confluent cultures are arrested at G1 or G2 phase, but not in M phase (S9 Fig, panel B). Hence, the observed effects of APH and CD437 on cccDNA synthesis under this experimental condition are unlikely due to their regulation of cell division. Taken together, several lines of evidence presented above strongly suggest that Pol α plays a rapid and direct role in cccDNA amplification in HepAD38 cells.

### Pol α and δ are recruited to HBV DNA

**Fig 6:**
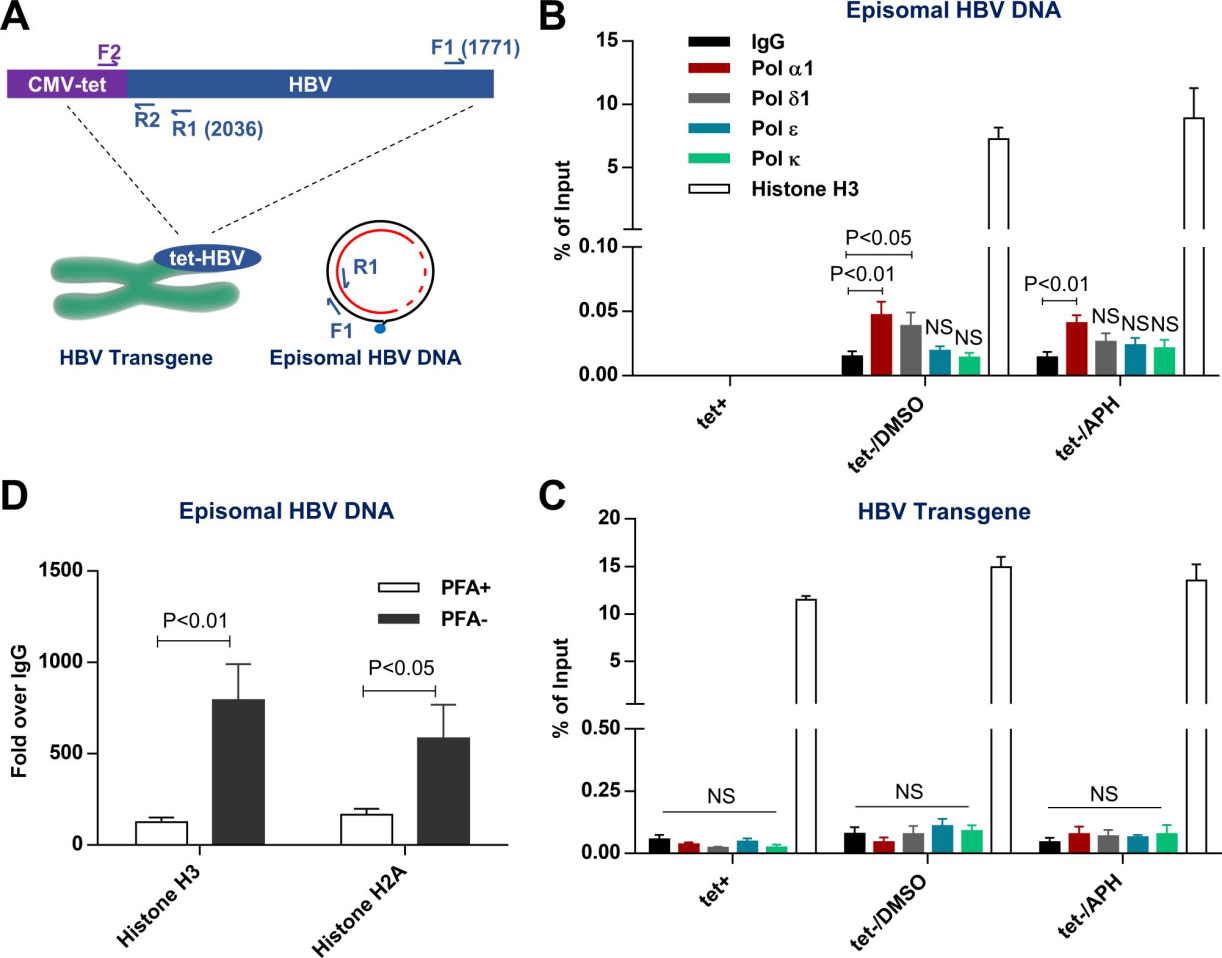
Pol α is associated with HBV DNA. (A) Schematic representation of 2 pairs of qPCR primers to differentially detect episomal HBV DNA (F1-R1) and integrated HBV transgene (F2-R2). (B-C) HepAD38 were either cultured in the presence of tet for 7 days, or in the presence of PFA from day 2 to day 6 after tet removal and followed by DMSO or 1 μM APH treatment during cccDNA formation from day 6 to day 7. Cells were harvested and analyzed by ChIP assay using antibodies against the indicated DNA polymerases. The enrichment of episomal HBV DNA (B) and HBV transgene (C) were measured by a qPCR assay. Rabbit IgG serves as a negative control. Histone H3 serves as a positive control. Data represent 4 independent experiments (mean ± SEM). Data are expressed as % of input and analyzed by two-tailed Student’s t-test (unpaired). (D) HepAD38 cells were cultured in the presence of PFA from day 2 to day 6 after tet removal. On day 6, PFA was either kept in or removed from culture media for another 24 h before harvesting and analyzing by ChIP assay using antibodies against histones H3 and H2A. Rabbit IgG serves as a negative control. The enrichment of HBV DNA was quantified by qPCR assay and presented as fold over IgG groups. Data represent 3 independent experiments (mean ± SEM). Data were analyzed by two-tailed Student’s t-test (unpaired).

As above functional studies have indicated that Pol α plays a direct role in cccDNA amplification, we further investigated whether Pol α and other B family DNA polymerases are physically recruited to HBV DNA during the conversion of rcDNA to cccDNA. To this end, we performed chromatin immunoprecipitation (ChIP) assay to characterize the potential interaction between HBV DNA and Polymerases, with histone H3 as a positive control. Because HepAD38 cell harbors a single copy of the tet-CMV IE promoter driven HBV transgene [28], we designed specific primers to selectively amplify episomal viral DNA (F1-R1) and the viral transgene integrated in chromosome (F2-R2) (Fig 6A). As anticipated, no episomal HBV DNA was pulled-down by the antibody against Pol α1 or histone H3 in the absence of HBV replication (Fig 6B, tet+). However, a significant association between episomal HBV DNA and Pol α1 or Pol δ, but not Pol ɛ and Pol κ, was detected at 24 h after removal of PFA to allow rcDNA and cccDNA synthesis to take place (Fig 6B, tet-/DMSO). Interestingly, inhibiting the conversion of rcDNA to cccDNA by APH did not alter the association of Pol α1 to episomal HBV DNA (Fig 6B, tet-/APH). This later result suggests that inhibition of Pol α1 enzymatic activity does not disrupt its recruitment to episomal HBV DNA. However, the sole scaffold function of Pol α1 devoid of enzymatic activity is not sufficient to support cccDNA synthesis. In marked contrast, only H3 antibody, but not antibody against any of the tested polymerases, could pull down HBV transgene in a manner independent of HBV DNA replication and cccDNA synthesis (Fig 6C). These results thus suggest that Pol α1 and Pol δ can be specifically recruited to HBV rcDNA, but not the HBV transgene integrated in the cellular chromosome, most likely through interacting with rcDNA associated proteins, such as other components of DNA repair machinery or recognition of specific histone post-translational modifications. Notably, because cccDNA constitutes only a small portion of total HBV DNA (Fig 1A) [26, 37], the fact that approximately 10% of episomal HBV DNA were associated with histone H3 indicates that in addition to cccDNA, other HBV nuclear DNA species, *i*.*e*. deproteinized (or protein-free) rcDNA, may also be associated with nucleosomes and exist as minichromosomes (Fig 6B). In support of this notion, blocking the conversion of DP-rcDNA to cccDNA by APH did not affect the association between histone H3 and HBV DNA (Fig 6B, tet-/APH). Moreover, histones H3 and H2A were significantly enriched onto episomal HBV DNA after enabling rcDNA synthesis by removal of PFA from culture medium (Fig 6D).

### Pol α is involved in cccDNA formation at a step prior to minus strand DNA ligation

**Fig 7:**
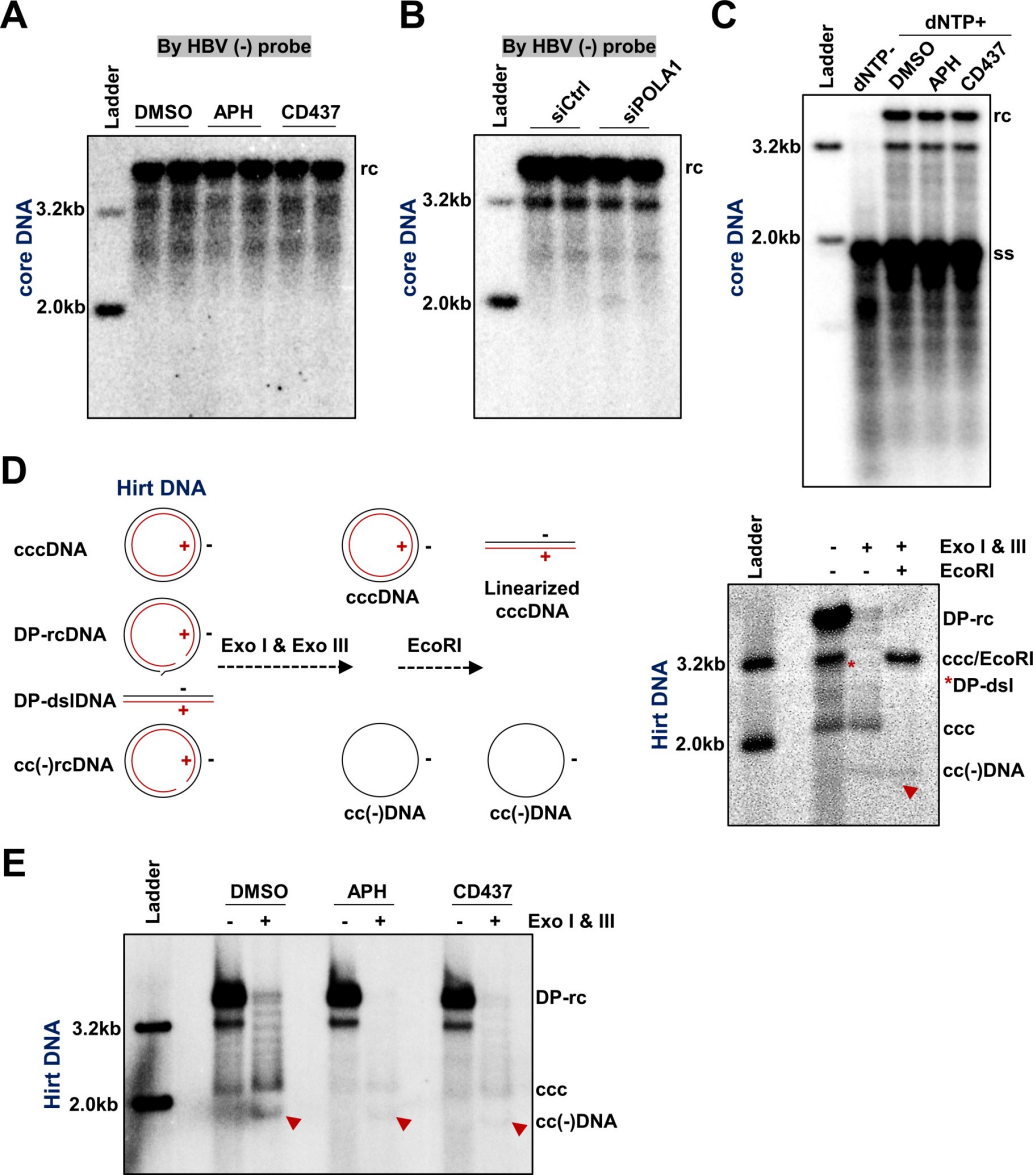
Pol α is required for the formation of covalently closed minus strand rcDNA. (A) HepAD38 cells were cultured in the presence of PFA from day 2 to day 6 after tet removal. From day 6, PFA was removed from culture media for 24 h in the presence of Pol α inhibitor APH (1 μM) or CD437 (1 μM), with DMSO as a control. Cytoplasmic HBV core DNA was extracted and analyzed by Southern blot hybridization by using HBV (-) probe to hybridize with viral plus strand. (B) HepAD38 cells were cultured in the presence of 2 mM PFA from day 2 to day 6 after tet removal. At day 4, the cells were re-seeded and transfected with 10 pmol siRNA targeting POLΑ1 or scramble siRNA, by using RNAiMAX. From 48 h to 72 h after siRNA transfection (day 6 to day 7 after tet removal), PFA was removed to resume viral DNA synthesis. Cytoplasmic HBV core DNA was extracted and analyzed by Southern blot hybridization by using HBV (-) probe to hybridize with viral plus strand DNA. (C) HepAD38 cells were cultured in the presence of PFA from day 2 to day 6 after tet removal, HBV intracellular nucleocapsids were purified by 30% sucrose cushion ultracentrifugation. *In vitro* EPR assay was performed at 37°C for 16 h in the absence or presence of Pol α inhibitor APH (1 μM) or CD437 (1 μM), with or without 0.1 mM of each dNTP. Upon completion of EPR reaction, viral DNA was extracted and analyzed by Southern blot hybridization. (D) HepAD38 cells were cultured in the absence of tet and HBV DNA replication was arrested by PFA treatment between day 2 to 6 after tet removal. The cells were then cultured in the presence of tet and absence of PFA for 24 h. Hirt DNA without prior treatment, treated with Exo I & III without or with following EcoRI digestion were resolved by agarose gel electrophoresis and detected by Southern blot hybridization with a minus-strand specific probe. (E) HepAD38 cells were cultured in the presence of PFA from day 2 to day 6 after tet removal. From day 6, cccDNA synthesis was initiated by removing PFA for 24 h in the presence of Pol α inhibitor APH (1 μM) or CD437 (1 μM), with DMSO as a control. Hirt DNA was extracted and analyzed by Southern blot hybridization without prior heat denaturation, or after Exo I & III digestion. Minus strand covalently closed DNA is denoted.

In order to determine which step of rcDNA to cccDNA conversion requires Pol α and other B group DNA polymerases, we first examined their role in the gap filling event (or the elongation) of viral plus strand DNA. To this end, HepAD38 cells were cultured in the absence of tet and presence of PFA for 4 days to arrest viral DNA synthesis at the full-length minus-strand DNA stage, and then were mock-treated (DMSO) or treated with 1 μM of APH or CD437 in the absence of PFA for 24 h to resume plus-strand DNA synthesis. HBV core DNA was detected by Southern blot assay with a probe specifically hybridizing to plus-stranded viral DNA. The results clearly indicated that both APH and CDC437 did not inhibit plus strand synthesis (Fig 7A). Similarly, siRNA knockdown of Pol α1 expression also did not affect plus strand synthesis (Fig 7B). In agreement with these results, *in vitro* synthesis of plus-strand DNA in purified PFA-arrested nucleocapsids in an endogenous DNA polymerase reaction (EPR) could not be inhibited by APH or CD437 (Fig 7C). These results clearly suggest that Pol α and other group B DNA polymerases do not play a role in elongation of the incomplete plus strand DNA in cccDNA synthesis. However, due to the limited resolution of this assay, the role of those cellular DNA polymerases in the closure of the gap or nick in both strands of rcDNA cannot be determined.

Recently, a covalently closed minus strand rcDNA, or cc(-)rcDNA, had been identified and considered as a potential intermediate of cccDNA synthesis [38, 39]. This finding suggests that the discontinuity in two strands of rcDNA is sequentially repaired. While the precursor—product relationship of DP-rcDNA, cc(-)rcDNA and cccDNA has not been firmly established in the field, it will be interesting to dissect their relationship by inhibition of cccDNA synthesis with DNA polymerase inhibitors. As shown in Fig 7D, treatment of Hirt DNA preparations with exonuclease I (Exo I) and III (Exo III) to remove DNA species containing open ends revealed a ladder of several DNA species migrating between supercoiled cccDNA and rcDNA, which can be converted into unit-length double-stranded linear DNA by EcoRI digestion and are thus different topological isoforms of supercoiled cccDNA [40, 41]. Moreover, an additional DNA species migrating faster than cccDNA and resistant to EcoRI digestion is the covalently closed minus strand DNA, or cc(-)DNA, which is derived from exonuclease digestion of the gapped positive strand of cc(-)rcDNA [38] (as depicted in Fig 7D). To investigate whether Pol α is required for the repair of the minus strand DNA gap in cccDNA amplification, HepAD38 cells were mock-treated or treated with 1 μM of APH or CD437 upon removal of PFA for 24 h. As anticipated, treatment with APH and CD437 reduced the levels of cccDNA. Interestingly, the level of cc(-)rcDNA was also reduced in APH and CD437 treated cells (Fig 7E). These results thus suggest that Pol α may play an important role in repairing the discontinuity of minus strand in the conversion of rcDNA to cccDNA.

## Discussion

Different from adenoviruses which antagonize cellular DNA repair machinery to prevent unwanted repair of its double-stranded linear DNA genome [42], or herpes simplex virus-1 (HSV-1) for which DNA repair proteins are recruited to their incoming genomes to restrict viral transcription [43], HBV, on the other hand, takes advantage of cellular DNA repair machinery to repair the discontinuity in its rcDNA genome and convert into a transcription permissive cccDNA. Indeed, due to its limited coding capacity, the replication of HBV genomic DNA heavily relies on the exploitation of host cellular proteins [44]. Several host cellular DNA repair proteins, including TDP2 [16], DNA ligases [18], FEN1 [17] and DNA topoisomerases [29] have been identified to be involved in cccDNA synthesis. Through a loss-of-function genetic screening in HBV infection of HepG2^NTCP^ cells, Pol κ and Pol λ were found to significantly contribute to cccDNA biosynthesis *in de novo* HBV infection [20]. Taking a chemical genetics approach, we demonstrated herein that both siRNA knockdown of Pol α expression and inhibition of its enzymatic activity by APH or CD437 reduced cccDNA intracellular amplification (Figs 1B, 2 and 3). The fact that cccDNA amplification in cells harboring mutant Pol α resistant to CD437 was no longer inhibited by the compound verified Pol α1 as its functional target in suppression of cccDNA synthesis (Fig 4). It is worth noting that our siRNA screening also identified Pol δ1 and Pol ε that appeared to contribute to cccDNA amplification (Fig 2). Moreover, reduced cccDNA synthesis in Pol δ knockout HepAD38 cells (S4 Fig) and recruitment of Pol δ to episomal HBV DNA (Fig 6B) strongly suggest an important role of this DNA polymerase in cccDNA amplification. Although Pol ε and Pol δ are primary DNA replication polymerases catalyzing leading chain and lagging chain elongation, they also play critical roles in nucleotide excision repair and base excision repair [31]. Hence, like Pol α, those DNA polymerases may also participate in cccDNA amplification

Interestingly, distinct from *de novo* cccDNA synthesis where Pol κ and Pol λ play an essential role [20], we found that these cellular DNA polymerases were dispensable for cccDNA intracellular amplification (Fig 2). This finding is consistent with the report showing that siRNA knockdown of Pol κ expression in HepDES19 cells did not inhibit cccDNA intracellular amplification [18]. Consistent with these functional assay results, ChIP assay also indicated that Pol κ was not recruited to episomal HBV DNA in HepAD38 cells (Fig 6B). Instead, we demonstrated herein that Pol α as well as Pol δ and ɛ play an important role in intracellular amplification of cccDNA but are not required for *de novo* cccDNA synthesis (Fig 1) [20]. The requirement of distinct cellular DNA polymerases in cccDNA synthesis through the two different pathways strongly suggests that different DNA repair complexes may be recruited to convert rcDNA from incoming virions and progeny nucleocapsids into cccDNA. One plausible explanation for this intriguing phenomenon is that whereas the plus-strand of rcDNA in virion particles is largely incomplete, the length of plus strand DNA from progeny mature nucleocapsid is much longer and close to completion [23, 37]. Such a structural difference in the plus strand 3’ terminus of precursor rcDNAs may result in the recruitment of different DNA repair complexes to catalyze their conversion into cccDNA. Alternatively, it is also possible that the infecting virions enter the cytoplasm of hepatocytes *via* endocytosis where the low pH environment in endosomes may trigger structural shifts of nucleocapsids. On the contrary, the intracellular progeny nucleocapsids are most likely not exposed to such an acid environment. The difference in nucleocapsid structures may result in nuclear import of rcDNA *via* distinct pathways and deposition of rcDNA in different regions of the nucleus. Indeed, a recent study revealed that interaction between capsid and a capsid disassembly related protein CPSF6 determines the nuclear domain where human immunodeficiency virus (HIV) DNA travels to and subsequently integrates at [45]. Moreover, human papilloma virus (HPV) DNA replication occurs in specific nuclear regions where fragile host DNA chromosomes and DNA repair proteins localize [46, 47]. Therefore, a likely scenario is that the distinct structure features presented on the nucleocapsids from incoming virions and intracellular progeny mature nucleocapsids direct their rcDNA to be transported to distinct nuclear domains where different DNA repair complexes are enriched and recruited for cccDNA synthesis. In addition, despite the fact that HBx protein is not required for *de novo* cccDNA synthesis [48], due to its expression in HBV replicating stable cell lines, such as HepAD38 and HepDES19 cells, but lack during the initial period of *de novo* infection, the contribution of HBx in the observed differential regulation of cccDNA amplification in these cell lines cannot be ruled out.

Mechanistically, our results suggest a direct role of Pol α in cccDNA amplification. *First*, inhibition of cccDNA amplification by Pol α inhibitors was rapid and independent of the induction of antiviral genes (5A, Fig 5C and 5E); *Second*, stopping new protein synthesis by cycloheximide did not attenuate the effect of Pol α inhibitors on cccDNA amplification (Fig 5D); *Third*, our experiments were conducted in confluent and quiescent cells, thereby the observed reduction in cccDNA synthesis by suppression of Pol α expression or inhibition of its polymerase activity is not due to the indirect effect of suppressing the cell cycle progression (S9 Fig). *Finally*, we demonstrated that Pol α1 and Pol δ are physically recruited to HBV rcDNA, but not to integrated HBV transgene, by ChIP assays (Fig 6).

It was reported recently that unintegrated retroviral DNA are loaded with histones quickly after their nuclear import to assemble into an extrachromosome structure that is under epigenetic regulation [49, 50]. Interestingly, we demonstrated herein that a substantial percentage (10%) of episomal HBV DNA is associated with histone H3 (Fig 6), suggesting that in addition to cccDNA, other HBV DNA species, most likely the nuclear DP-rcDNA, may also acquire nucleosome structures and exist as episomal minichromosomes [51]. This notion is further supported by the results that inhibition of rcDNA conversion into cccDNA by APH does not alter the amount of HBV DNA associated with histones. Moreover, accumulating evidence suggests that certain histone modifications that orchestrate nucleosome remodeling at DNA damage sites are involved in regulating the recruitment of DNA repair factors [52–55]. Therefore, histone association or chromatinization of rcDNA may be necessary for efficient recruitment of DNA repair complex and cccDNA formation. Compared to histones, only a small, but significant fraction of episomal HBV DNA (approximately 0.05%) were associated with Pol α1 or δ, which is consistent with the fact that only a small fraction of DP-rcDNA is converted into cccDNA in hepatoma cells. Although Pol α can bind to histone H2A and histone H2B *in vitro* [56], its differential recruitment to a small fraction of HBV episomal rcDNA, but not to the integrated HBV transgene, indicated that other viral and/or host cellular factors play a role in recruitment of Pol α to HBV DNA.

In addition to rcDNA, a small percentage of cccDNA is converted from dslDNA, a replication intermediate generated through *in-situ* priming of plus strand DNA synthesis [57], *via* non-homologous end joining (NHEJ) DNA repair pathway [27, 58]. The dslDNA-derived cccDNA usually does not support productive viral replication due to the error-prone repair of viral DNA by the NHEJ pathway [59, 60]. As stated above, despite the fact that several DNA repair enzymes have been identified to play essential roles in cccDNA synthesis from rcDNA precursor, the DNA repair pathways that catalyze rcDNA from either incoming virions or mature progeny nucleocapsids into cccDNA remain elusive. However, the recent identification of a cc(-)rcDNA species suggests that conversion of rcDNA into cccDNA might be through a sequential repair of the nick and gap of minus and plus strands of rcDNA by one or two distinct single-strand DNA repair complexes [17, 38]. The fact that inhibition of Pol α by CD437 reduced the formation of cc(-)rcDNA (Fig 7E) indicates that Pol α is required for the repair of rcDNA minus strand. Recently, FEN1, a single strand DNA repair component, had been shown to be required for cccDNA synthesis *via* both *de novo* infection and intracellular amplification pathways, most likely by processing the 5’ flapped structure presented in rcDNA. It will be interesting to further investigate the functional relationship between Pol α and FEN1 in cccDNA synthesis. Unlike its role in DNA replication, the role of Pol α in DNA repair was only intensively studied recently. Particularly, in addition to promoting NHEJ DNA repair in Hela cells [61], Pol α has been shown to catalyze the fill-in synthesis to counteract DNA hyper-resection in double strand break repair of telomerase DNA and hence, regulate the choices of DNA repair pathways [62, 63]. Since our results suggest a role of Pol α in the repair of the HBV minus strand DNA discontinuity (Fig 7E), it is possible that Pol α catalyzes the partial extension of the 3’ end in the minus strand that creates a homology overlap with the 5’ end, which could be repaired through the homology-directed DNA repair pathway. This hypothesis will be investigated by comparing the terminal sequence of minus strand DNA bound to Pol α in the absence and presence of APH by ChIP-seq technology.

In conclusion, taking advantage of a chemical genetics screening approach in combination with our synchronized and rapid cccDNA formation assay in HepAD38 cells, we obtained evidence supporting a hypothesis that cellular Pol α as well as other members of B group DNA polymerases are required for cccDNA intracellular amplification, most likely through direct recruitment to nuclear rcDNA and catalyzing cccDNA synthesis. The cccDNA intracellular amplification in human and mouse hepatocyte-derived cell lines supporting transient or stable HBV replication or in the liver of HBV transgenic mice have been documented in the last three decades [26, 28, 64–67] and widely used as the model of HBV cccDNA synthesis [17, 18, 38]. However, due to the small extent or undetectable level of cccDNA amplification in HBV infected primary human hepatocytes or NTCP-expressing HepG2 cells [20, 22, 48, 68, 69], it remains to be determined whether the cccDNA amplification in the stable cell lines and HBV infected hepatocytes is via the same or distinct mechanisms. Nevertheless, identification of distinct cellular DNA polymerases required for *de novo* cccDNA synthesis and intracellular cccDNA amplification sheds light on uncovering the DNA repair pathways governing the establishment and maintenance of cccDNA pools in HBV infected hepatocytes, which will establish molecular basis for development of novel therapeutics to resolve chronic HBV infections [44].

## Materials and methods

### Cell culture

Human hepatoblastoma cell HepG2 and its subclone C3A (ATCC HB-8065) were purchased from ATCC. C3A^hNTCP^ cell line stably expressing human NTCP was established as previously described [70]. HepG2 and C3A^NTCP^ were cultured in DMEM/F12 media (Corning) supplemented with 10% fetal bovine serum (FBS), 100 U/ml penicillin and 100 μg/ml streptomycin. HepAD38 is an HepG2 derived cell line that supports tetracycline (tet)-off inducible HBV replication and was provided by Dr. Christoph Seeger at Fox Chase Cancer Center [28]. HepDES19 is an HepG2 derived cell line supporting tet-off inducible replication of HBV with deficiency of envelope protein expression and was established in our laboratory [26]. HepAD38 and HepDES19 cells were maintained in DMEM/F12 media supplemented with 10% FBS, 100 U/ml penicillin, 100 μg/ml streptomycin, 1 μg/ml tet and 400 μg/ml G-418. Tet was removed from HepAD38 or HepDES19 culture media when initiation of HBV replication is needed. All cells were maintained in a 5% CO_2_ incubator at 37°C. All cell culture experiments were performed in 50 μg/ml rat tail collagen (Corning) coated plates.

### Antibodies, chemicals and plasmids

Anti-Pol α1 antibody (sc-373884), anti-Pol δ1 antibody (sc-17776) and anti-Pol α2 antibody (sc- 398255) used in Western blot assays were purchased from Santa Cruz Biotechnology and used with 1:200 dilution. Anti-β-actin antibody (3700) was purchased from Cell Signaling Technology and used with 1:2000 dilution. Anti-Pol α1 antibody (ab177994), anti-Pol δ1 antibody (ab225907) and anti-Pol ε antibody (ab241943) used in chromatin immunoprecipitation assays were purchased from Abcam. Anti-Pol κ antibody was purchased from Bethyl Laboratories (A301-977A). Anti-histone H3 (4620) and anti-histone H2A (12349) antibodies were purchased from Cell Signaling Technology. Anti-rabbit IgG was purchased from Sigma (31887). All the antibodies used in chromatin immunoprecipitation are IP compatible. Foscarnet (P6801), aphidicolin (A0781) and CD437 (C5865) were purchased from Millipore-Sigma. Cycloheximide (2112) was purchased from Cell Signaling Technology. Myrcludex B is a gift of Dr. Stephan Urban [71]. Entecavir (ETV) was provided by Dr. Willan S. Mason at Fox Chase Cancer Center. pLX-sgRNA was a gift from Eric Lander & David Sabatini (Addgene 50662). pcDNA3.1/POLD1-FLAG plasmid was purchased from Genscript (OHu14862).

### A synchronized and rapid cccDNA synthesis assay in HepAD38 cells

HepAD38 cells were seeded into 6-well plates at a density of 6 × 10^5^ cells per well and cultured in the absence of tet to initiate HBV replication. Two days later, 2 mM PFA was added into the culture media to arrest and synchronize HBV replication. Culture media were refreshed every other day. On day 6 post seeding, 1 μg/ml tet was added into culture medium to stop pgRNA transcription from HBV transgene and PFA was removed from culture medium to resume HBV DNA synthesis and cccDNA formation. Effects of compounds on cccDNA synthesis can be evaluated by treating the cells starting at PFA removal for 24 h. To test the effect of compounds on cccDNA stability, the cells were left untreated after PFA removal for 48 h to allow the establishment of cccDNA pool, followed by compound treatment at desired concentrations for 24 h in the presence of 1 μM Entecavir. Hirt DNA was extracted and amounts of cccDNA were determined by Southern blot hybridization as described below.

### siRNA transfection

HepAD38 cells were seeded into 6-well plates at a density of 6 × 10^5^ cells per well and cultured in the absence of tet. Two days later, 2 mM PFA was added into the culture media. On day 4 post seeding, the cells were re-seeded and transfected with 10 pmol siRNA oligos and 1 μl RNAiMAX (life technologies) following the manufacturer’s protocol. Two days later, 1 μg/ml tet was added into culture medium to stop pgRNA transcription from HBV transgene and PFA was removed from culture medium to resume HBV DNA synthesis and cccDNA formation. Cells were harvested 24 h later. Total RNA was extracted using TRIzol reagent (Invitrogen). Gene silencing efficiencies were validated by qRT-PCR (comparative Ct method (ΔΔCt) using β-actin as an internal control) or Western blot assays. The siRNA oligo sequences for screening are listed in S1 Table. The qRT-PCR primers are listed in S2 Table. The Pol α1 siRNA (SR303612) and Pol α2 siRNA (SR308429) used in validation experiments (Fig 4A and S6 Fig) were purchased from Origene.

### Production of HBV virions and infection of C3A^hNTCP^ cells

HBV virions were harvested from culture media of HepAD38 cells and concentrated by 8% PEG-8000 as described previously [70]. For infection, C3A^hNTCP^ cells were cultured in DMEM supplemented with 3% FBS, and 2% DMSO for 24 h. The cells were then infected with HBV at a MOI of 500 genome equivalents in DMEM containing 3% FBS, 2% DMSO and 4% PEG-8000 (Sigma P1458). The inoculums were removed at 12 h post infection (hpi) and the cell monolayers were washed with PBS for 5 times before refreshing with DMEM containing 3% FBS, 2% DMSO and 4% PEG-8000. The infected cultures were harvested at 12 or 48 hpi.

### Establishment of DNA *POLD1* knockout cell lines

The *POLD1* sgRNA CRISPR-Cas9 All-in-One Lentivector was purchased from Abm Biology (target sequence: CGAGGATCTATGGCTGATGG). Lenti-X Packaging Single Shots (VSV-G) (Clontech) were used to package *POLD1* sgRNA CRISPR-Cas9 virus following manufacturer’s protocol. One milliliter of *POLD1* sgRNA CRISPR-Cas9 virus preparation mixed with 1 ml DMEM/F12 complete media containing 1 μg/ml tet were applied to HepAD38 cells. After selection with 2 μg/ml puromycin for 2 weeks, the survival cell clones were expanded. *POLD1* knockout was verified by Western blot assay and Sanger sequencing of genomic DNA in a single clone and designated as HepAD38-*POLD1*^-/-^.

### CRISPR knock-in to generate HepAD38-Cas9-*POLΑ1*^L764S^

This gene editing protocol was modified from its original designer [33]. At first, Cas9-expressing lentivector (Dharmacon) was packaged into lentivirus by using Lenti-X Packaging Single Shots (VSV-G) (Clontech) following the manufacturer’s protocol. HepAD38 cells were transduced with the Cas9-expressing lentivirus and selected by 10 μM Blasticidin for 2 weeks to acquire HepAD38-Cas9 cells. Secondly, *POLA1* sgRNA sequence 5’-GTTAGTGATCTGCAATGCTAA-3’ was cloned into pLX-sgRNA vector (Addgene 50662), resulting in pLX-sgPOLΑ1 that expresses guide RNA targeting *POLA1* L764 coding region. To knock-in *POLΑ1*^L764S^, 1 μg of pLX-sgPOLΑ1 plasmid were transfected into 1 × 10^6^ HepAD38-Cas9 cells seeded in a 6-well plate, in combination with 50 pmol synthesized single-stranded DNA oligo 5’-ACCCCAAGCAAACACTGAATCCAACAGGAAATGCTTTTTCCCCCTTTCTAAGTTAAATTTACCATAATGTTCCCAGCGATGTTAGTGATCTGCAATGCTGATGGAAGAACATTTAGCTCACACATGATCTGCAAAATGAACTTGGCATCTTTCCAGGTGTGTTCCAACAGGTATAACAGTTGAGAAGATTCACTGTACAG-3’ serving as a repair template, by using lipofectamine 2000 according to the manufacturer’s protocol. Two days after transfection, cells were exposed to 10 μM CD437 for 14 days and the surviving single colonies were expanded into cell lines. To validate *POLΑ1*^L764S^ knock-in, genomic DNA from the isolated cell lines was extracted and DNA flanking L764 coding region was amplified by PCR with 5’- AGCATTGGGATCAGTGGTATG-3’ and 5’-AACACGCTGCACCTGGCATTC-3’ primer pair, and L764S mutation were confirmed by Sanger sequence method.

### Southern blot analysis of HBV DNA

HBV cccDNA was extracted by a modified Hirt DNA extraction protocol [37]. Briefly, cells from one well of a 6-well plate were lysed by 800 μl Hirt DNA lysis buffer (10 mM Tris-HCl, pH 8.0; 0.625% SDS; 10 mM EDTA) for 30 min at room temperature, followed by adding 200 μl of 5 M NaCl to thoroughly mix and incubate overnight at 4°C. On the next day, supernatants were collected after centrifugation at 12000 × g for 30 min at 4°C, phase extracted by phenol for twice and phenol-chloroform (1:1) for once. DNA in the aqueous phase was precipitated by mixing with 0.7 volume of isopropanol at -20°C overnight. DNA was pelleted by centrifugation at 12,000 × g, washed by 70% ethanol and dissolved in 20 μl nuclease-free water. If indicated, Hirt DNA samples were denatured at 88ºC for 5 minutes and chilled on ice. Such a procedure allows the complete denaturation of DP-rcDNA into single-stranded DNA, whereas cccDNA remains as a double-stranded circular DNA. The heat denatured Hirt DNA samples were further digested with EcoRI to linearize cccDNA into unit-length double-stranded linear DNA. If needed, Hirt DNA was digested by Exo I & III to reveal minus strand covalently closed rcDNA.

Intracellular HBV core DNA was extracted by using 400 μl of core DNA lysis buffer (10 mM Tris-HCl, pH 8.0; 1 mM EDTA; 1% Nonidet P-40) to lyse cells from one well of a 12-well plate for 10 min at room temperature. Cytoplasmic fraction was acquired after spinning at 12,000 × g to remove cell debris, and further subjected to 200 μg/ml proteinase K (Ambion) digestion in proteinase K digestion buffer (10 mM Tris-HCl, pH 8.0; 100 mM NaCl; 1 mM EDTA; 0.5% SDS) for 1 h at 45°C. Equal volume of phenol-chloroform was used to perform phase extraction. DNA in the aqueous phase was then extracted following the same procedure described above for Hirt DNA.

For Southern blot analysis, the extracted DNA samples were resolved in 1.2% agarose gel electrophoresis and transferred onto an Amersham Hybond-N+ membrane (GE Healthcare). After UV crosslink, the membrane was probed with α-^32^P-UTP labeled plus-strand specific full-length riboprobe. α-^32^P-UTP labeled minus-strand specific full-length riboprobe was used if indicated (Fig 7A and 7B). Radioactive signals were imaged by a Typhoon scanner.

### Chromatin immunoprecipitation

Approximately 2 × 10^7^ cells in a 10 cm Petri dish were crosslinked by 1% formaldehyde for 10 min at room temperature, followed by quenching with 0.125 M glycine for 5 min. Cells were then lysed on ice for 10 min in 1 mL ChIP lysis buffer (50 mM Tris-HCl, pH 8.0; 1% SDS; 10 mM EDTA) containing 1 × protease inhibitor cocktail (Roche), scraped and collected into a 1.5 mL tube followed by ultrasound sonication to produce average fragment size 500–1000 bp of DNA. For each immunoprecipitation reaction, 100 μl of the supernatant (25 μg chromatin DNA) were used to incubate with 1 μg indicated antibody in ChIP dilution buffer (10 mM Tris-HCl, pH 8.0; 1% Triton X-100; 0.1% SDS; 150 mM NaCl; 2 mM EDTA) containing 1 × protease inhibitor cocktail (Roche) overnight at 4°C. Next day, 25 μl of protein G Dynabeads (Invitrogen) were added to each reaction and incubated for another 6 h at 4°C. Beads were then washed twice by ChIP low salt buffer (20 mM Tris-HCl, pH 8.0; 1% Triton X-100; 0.1% SDS; 150 mM NaCl; 2 mM EDTA), ChIP high salt buffer (20 mM Tris-HCl, pH 8.0; 1% Triton X-100; 0.1% SDS; 500 mM NaCl; 2 mM EDTA), ChIP LiCl buffer (10 mM Tris-HCl, pH 8.0; 1% Nonidet P-40; 250 mM LiCl; 1 mM EDTA) and TE buffer (10 mM Tris-HCl, pH8.0; 1 mM EDTA); and eluted by 200 μl ChIP elution buffer (TE buffer containing 1% SDS; 100 mM NaCl; 5 mM dithiothreitol). ChIP elutes were incubated at 65°C overnight to reverse crosslink Protein-DNA complex. After digestion with 1 μl of 1 μg/ml RNase A (Roche) at 37°C for 1 h followed by 2 μl of 20 mg/ml proteinase K at 37°C for another 2 h, DNA was purified by Qiagen DNA purification kit and stored at -20°C. DNA quantity was measured by real time PCR using specific primers listed in S2 Table.

### Endogenous Polymerase Reaction (EPR)

HepAD38 cells were cultured in tet-free media for 6 days with 2 mM PFA added from day 2 to day 6 to arrest HBV DNA replication. After harvesting, one 10 cm dish of cells were lysed with 3 ml core DNA lysis buffer (10 mM Tris-HCl, pH 8.0; 1 mM EDTA; 1% Nonidet P-40) for 10 min at room temperature, followed with 4 h 45,000 × rpm ultracentrifugation in 30% sucrose at 4°C to pellet intracellular capsids. After resuspending in 300 μl of TNE buffer (0.15 M NaCl; 0.01 M Tris-HCl, pH 7.4; 0.1 mM EDTA), each 50 μl of the aliquots were subjected to incubation with 50 μl 2 × EPR buffer (0.15 M NaCl; 0.1 M Tris-HCl, pH 8.0; 20 mM MgCl_2_; 2 mM dithiothreitol; 0.2% (vol/vol) Nonidet P-40) with indicated compound treatment for 16 h at 37°C. The next day, the reaction mix was subjected to core DNA extraction and the extracted DNA samples were resolved by agarose gel electrophoresis and HBV DNA was detected by Southern blot hybridization.

### Flow cytometry assay

HepAD38 cells were cultured in 6-well plates at sub-confluent (50%) or confluent (100%) conditions and then treated with the indicated compounds (DMSO, 1 μM APH, 1 μM CD437). After compound treatment for 24 h, cells were fully trypsinized into individual single cells and fixed with 66% ethanol overnight at 4°C. Next day, cells were stained with 1 × Propidium iodide staining buffer (Abcam 139418) containing 10 μg/ml Propidium Iodide solution and 500 U/ml RNaseA at 37ºC in the dark for 30 minutes. Approximately 1,000 cells/sample were analyzed using flow a BD FACS Canto with excitation laser at 488nm and emission detected using detector D with 575/25 bandpass filter. The flow histograms were generated using FlowJo software.

## Supporting information

S1 Fig A fast and synchronized cccDNA synthesis assay in HepAD38 cells. HepAD38 cells were cultured in the presence of 2 mM PFA to arrest HBV replication from day 2 to day 6 after tet removal. On day 6, cccDNA synthesis was either kept arrested by PFA or resumed by removing PFA. Cells were harvested at the indicated time post PFA removal. Cytoplasmic HBV core DNA and Hirt DNA were extracted and detected by Southern blot assays, with mtDNA as a loading control of Hirt DNA. The intensity of cccDNA band at each time point was quantified by ImageJ and presented as the relative amount over that in cells harvested at 0 h of PFA removal.
(TIF)

S2 Fig CD437 and APH reduce HBV cccDNA intracellular amplification in HepDES19 cells. HepDES19 cells were cultured in the presence of 2 mM PFA to arrest HBV replication from day 2 to day 6 after tet removal. On day 6, cccDNA synthesis was allowed by removing PFA for 24 h and treated with the indicated concentrations of CD437 and APH. Cytoplasmic HBV core DNA and Hirt DNA were extracted and detected by Southern blot assays, with mtDNA as a loading control of Hirt DNA.
(TIF)

S3 Fig The effect of APH on cccDNA formation is independent of PFA arresting of viral DNA replication. HepAD38 cells were cultured in tet-free media for 6 days followed with 48-hour treatment of DMSO, 1 μM APH or 1 μM ETV in the presence of tet. ETV is a viral polymerase inhibitor that stops viral DNA synthesis. Cytoplasmic HBV core DNA and Hirt DNA were extracted and detected by Southern blot hybridization, with mtDNA as a loading control of Hirt DNA.
(TIF)

S4 Fig DNA polymerase δ contributes to cccDNA amplification and may, at least in part, mediate APH inhibition of cccDNA synthesis. (A) The expression of Pol δ1 and β-actin in HepAD38 and HepAD38-*POLD1*^-/-^ cells were determined by Western blot assays. (B) *POLD1* guide RNA targeting sequence was presented. The guide RNA targeting region of *POLD1* was PCR amplified from genomic DNAs of both wild-type and *POLD1*^-/-^ cells, and sequence alignment was presented. (C) HepAD38 and HepAD38-*POLD1*^-/-^ cells were cultured in the presence of 2 mM PFA from day 2 to day 6 after tet removal. From day 6, cccDNA synthesis was initiated by removing PFA for 24 h in the presence or absence of 1 μM APH. Cytoplasmic HBV core DNA and Hirt DNA were extracted and detected by Southern blot hybridization, with mtDNA as a loading control of Hirt DNA. (D) HepAD38 and HepAD38-*POLD1*^-/-^ cells were cultured in the presence of PFA from day 2 to day 6 after tet removal. At day 4, the cells were re-seeded in 6-well plate and transfected with 2 μg of pcDNA3.1/POLD1-FLAG plasmid, using lipofectamine 3000. From 48 h to 72 h after plasmid transfection (day 6 to day 7 after tet removal), PFA was removed to allow cccDNA synthesis. The cells were harvested at 72 h after transfection, and protein levels of Pol δ1 and β-actin were determined by Western blot assays. Hirt DNA was extracted and HBV DNA was detected by Southern blot analysis, with mtDNA as a loading control.
(TIF)

S5 Fig Knock down of Pol α1 does not completely abolish the effect of APH on cccDNA synthesis. HepAD38 cells were cultured in the presence of 2 mM PFA from day 2 to day 6 after tet removal. At day 4, the cells were re-seeded and transfected with 10 pmol siRNA targeting POLA1 or with scrambled siRNA by using RNAiMAX. From 48 h to 72 h after siRNA transfection (day 6 to day 7 after tet removal), cccDNA synthesis was resumed by removal of PFA from culture medium, followed by DMSO or 1 μM APH treatment. The cells were harvested at 72 h after siRNA transfection, and protein levels of Pol α1 and β-actin were determined by Western blot assays. Cytoplasmic HBV core DNA and Hirt DNA were extracted and detected by Southern blot assays, with mtDNA as a loading control of Hirt DNA. The intensity of HBV cccDNA band was quantified by ImageJ and presented as relative amount in comparison with that in cells transfected with scramble siRNA and treated with DMSO.
(TIF)

S6 Fig Dissecting the function of primase complex subunits in cccDNA synthesis. (A) HepAD38 cells were cultured in the presence of PFA from day 2 to day 6 after tet removal. At day 4, the cells were re-seeded and transfected with 10 pmol siRNA targeting POLΑ1, POLA2 or scramble siRNA by using RNAiMAX. From 48 h to 72 h after siRNA transfection (day 6 to day 7 after tet removal), PFA was removed to allow cccDNA synthesis. The cells were harvested at 72 h after siRNA transfection, and protein levels of Pol α1, Pol α2 and β-actin were determined by Western blot assays. Cytoplasmic HBV core DNA and Hirt DNA were extracted and detected by Southern blot analysis, with mtDNA as a loading control of Hirt DNA. (B) The intensity of HBV cccDNA bands was quantified by ImageJ and presented as relative amount in comparison with that in cells transfected with scramble siRNA. Data represent 3 independent experiments. Data were analyzed by two-tailed Student’s t-test (unpaired), *P* < 0.001.
(TIF)

S7 Fig CD437 treatment does not affect cccDNA stability. HBV cccDNA pool was allowed to be established for 48 h after removal of PFA and addition of tet on day 6. Cells were then treated with indicated concentrations of CD437 for another 24 h. Hirt DNA was extracted and HBV DNA was detected by Southern blot hybridization, with mtDNA as a loading control.
(TIF)

S8 Fig Different clones harboring single amino acid mutation of Pol α abolish CD437 inhibition of cccDNA synthesis. HepAD38, HepAD38-Cas9 and 6 independent clones derived from HepAD38-Cas9 harboring single amino acid mutation of Pol α namely HepAD38-*POLΑ1*^L764S^ C2 C3 C4 C5 C6 and C7 were cultured in the presence of 2 mM PFA from day 2 to day 6 after tet removal. On day 6, cccDNA synthesis was initiated by removing PFA for 24 h in the presence of 1 μM CD437 treatment. Hirt DNA was extracted and HBV DNA was detected by Southern blot assays with mtDNA as a loading control.
(TIF)

S9 Fig The reduction of cccDNA amplification by Pol α inhibitors is independent of altering cell cycle. HepAD38 cells at sub-confluent (50%) or confluent (100%) conditions were treated with indicated compounds and harvest after 24 h. (A) Cell cycle stages were determined by flow cytometry with proprium iodide (PI) staining for DNA content. The y-axis indicates cell counts and the x-axis indicates propidium iodide fluorescence intensity. (B) Protein levels of p-Histone H3 (Ser10) and total Histone H3 were determined by Western blot assays.
(TIF)

S1 Table Sequence of siRNAs used in the screening of cellular DNA polymerases required for HBV cccDNA amplification. (DOCX)

S2 Table Sequence of primers used in qPCR analysis of cellular and viral RNA and DNA. (DOCX)
