# Supplementary figures and images for: DNA Polymerase alpha is essential for intracellular amplification of hepatitis B virus covalently closed circular DNA
Source: PLoS Pathog. 2019 Apr 26;15(4):e1007742. doi: 10.1371/journal.ppat.1007742 (PMC6505960; doi:10.1371/journal.ppat.1007742)

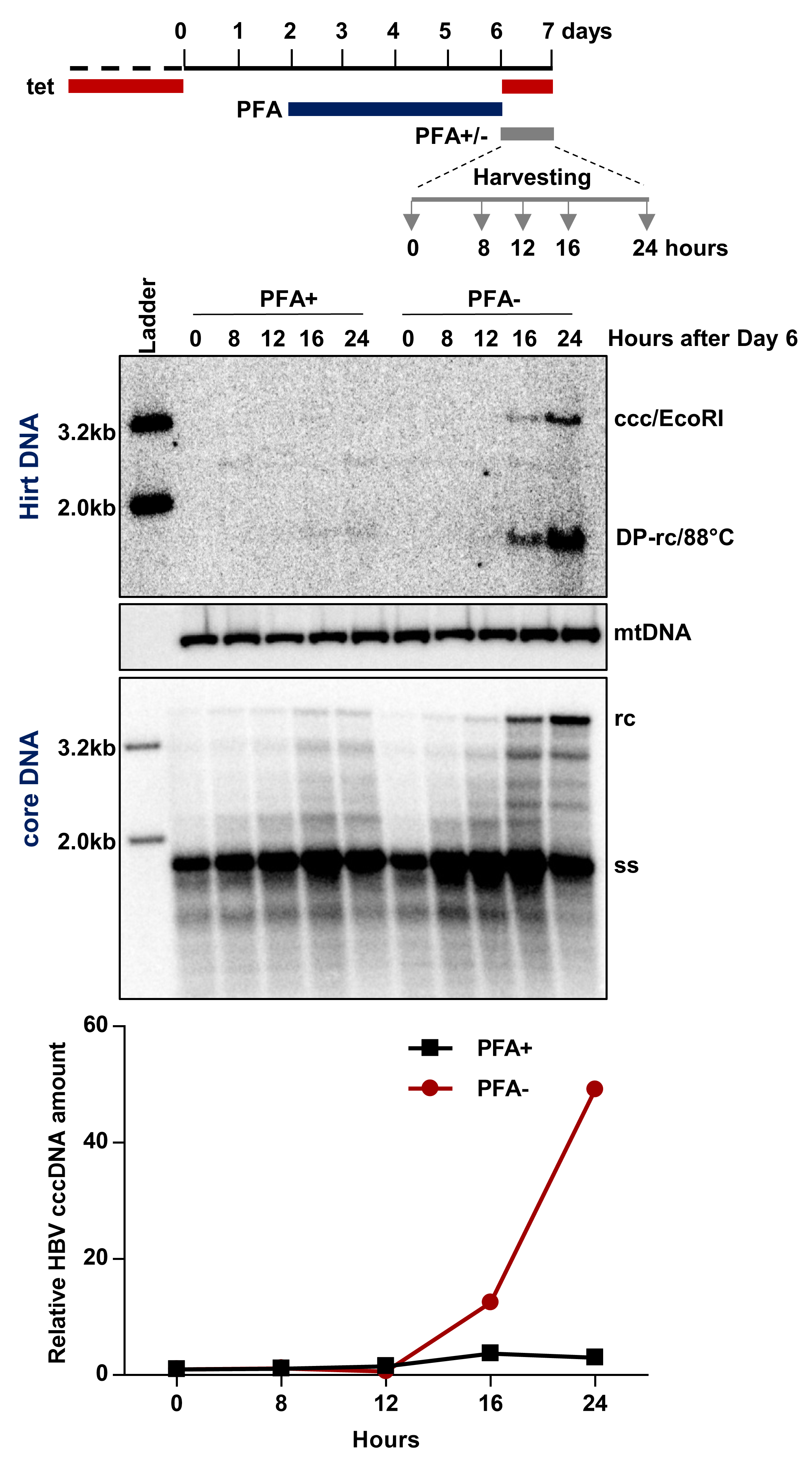

Supplement: S1 Fig — HepAD38 cells were cultured in the presence of 2 mM PFA to arrest HBV replication from day 2 to day 6 after tet removal. On day 6, cccDNA synthesis was either kept arrested by PFA or resumed by removing PFA. Cells were harvested at the indicated time post PFA removal. Cytoplasmic HBV core DNA and Hirt DNA were extracted and detected by Southern blot assays, with mtDNA as a loading control of Hirt DNA. The intensity of cccDNA band at each time point was quantified by ImageJ and presented as the relative amount over that in cells harvested at 0 h of PFA removal. (TIF) [file ppat.1007742.s001.tif]

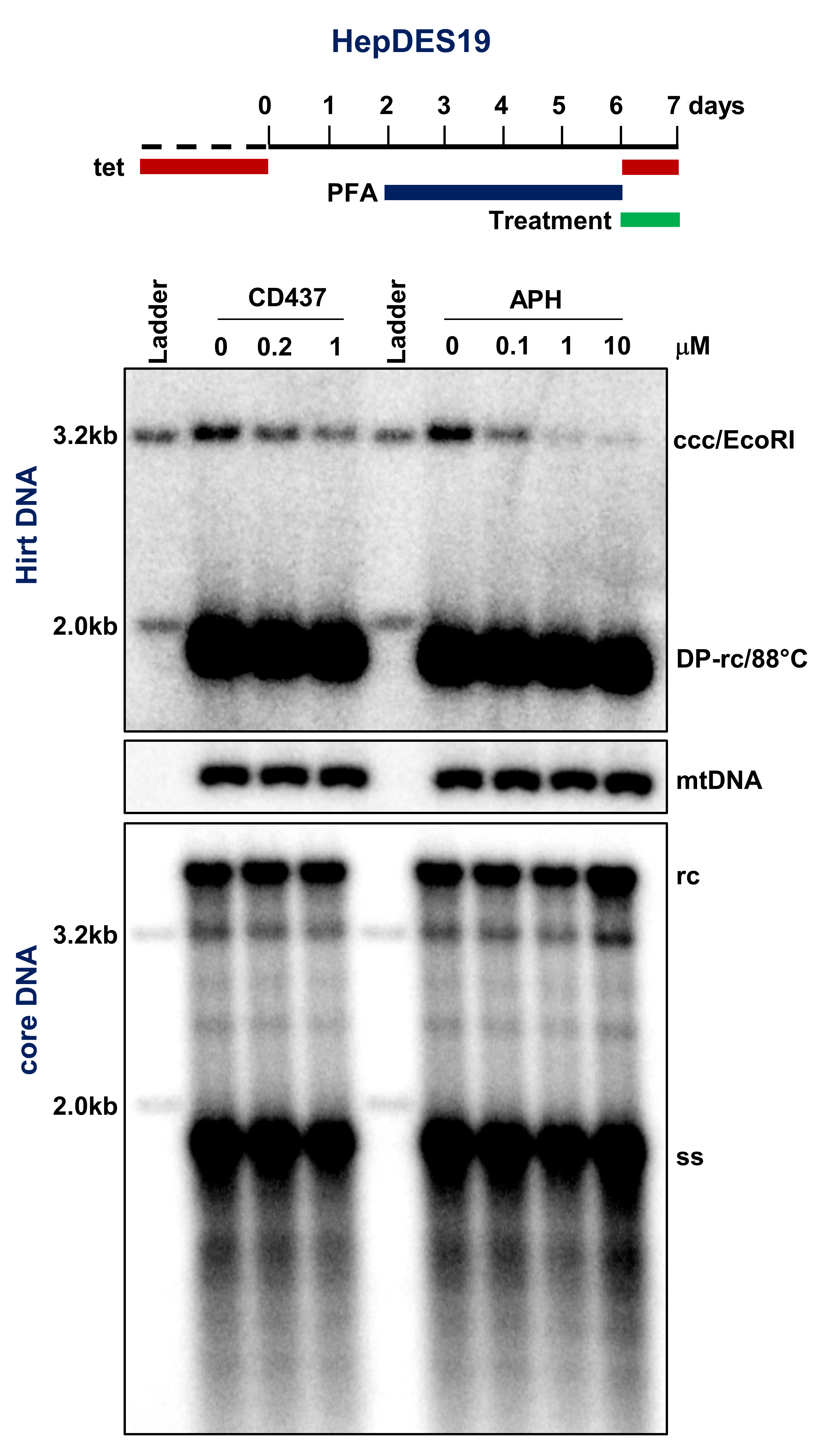

Supplement: S2 Fig — HepDES19 cells were cultured in the presence of 2 mM PFA to arrest HBV replication from day 2 to day 6 after tet removal. On day 6, cccDNA synthesis was allowed by removing PFA for 24 h and treated with the indicated concentrations of CD437 and APH. Cytoplasmic HBV core DNA and Hirt DNA were extracted and detected by Southern blot assays, with mtDNA as a loading control of Hirt DNA. (TIF) [file ppat.1007742.s002.tif]

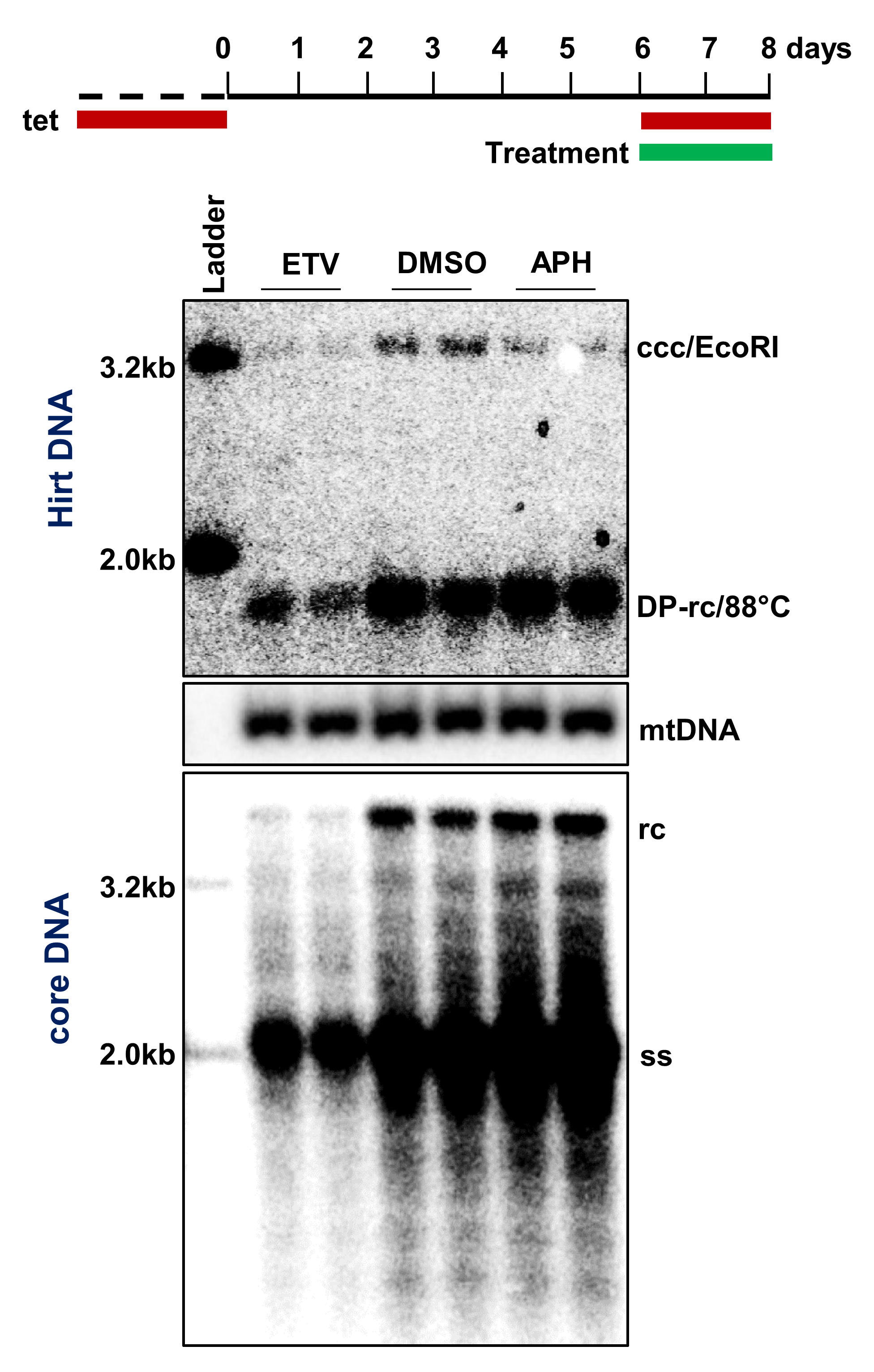

Supplement: S3 Fig — HepAD38 cells were cultured in tet-free media for 6 days followed with 48-hour treatment of DMSO, 1 μM APH or 1 μM ETV in the presence of tet. ETV is a viral polymerase inhibitor that stops viral DNA synthesis. Cytoplasmic HBV core DNA and Hirt DNA were extracted and detected by Southern blot hybridization, with mtDNA as a loading control of Hirt DNA. (TIF) [file ppat.1007742.s003.tif]

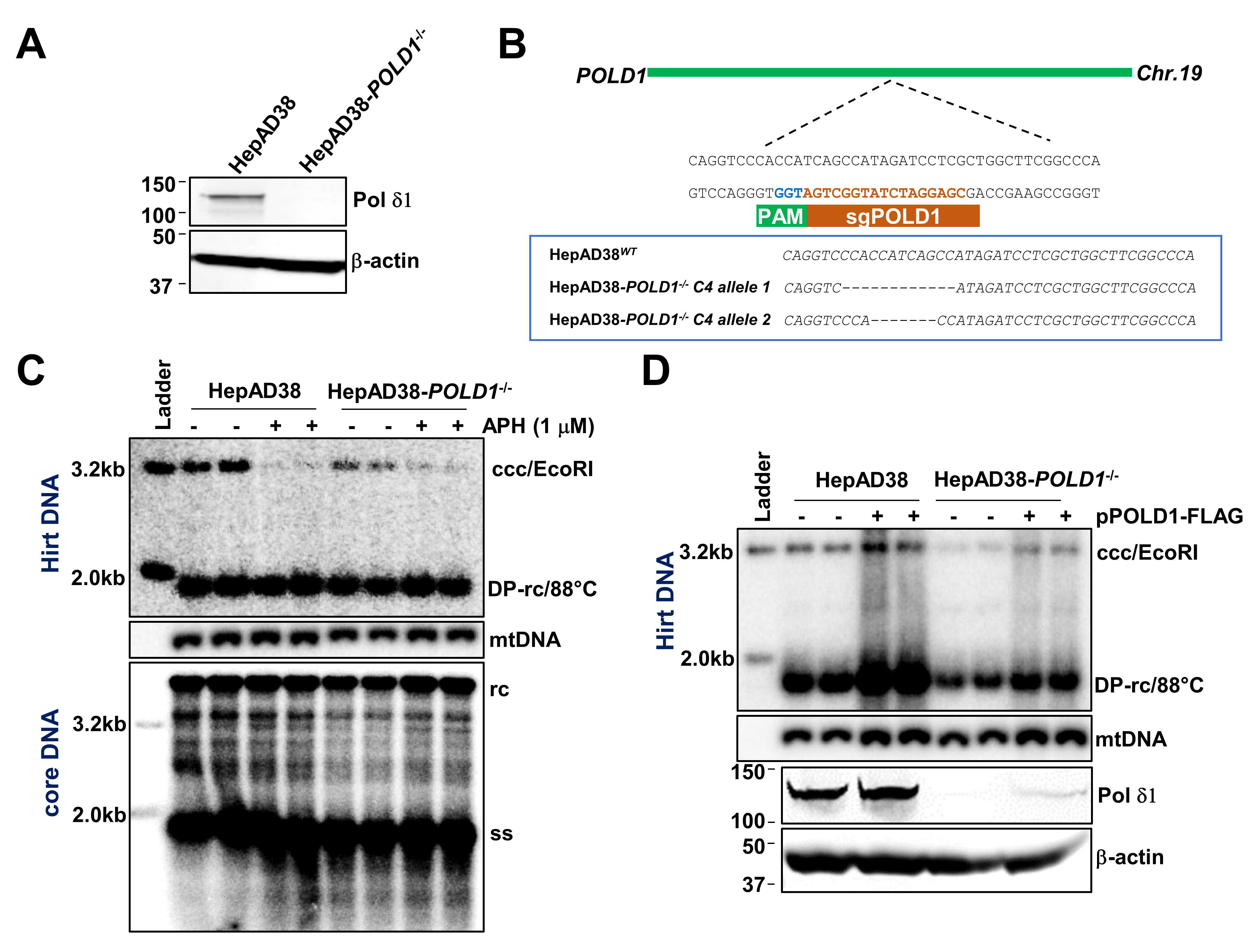

Supplement: S4 Fig — (A) The expression of Pol δ1 and β-actin in HepAD38 and HepAD38-POLD1-/- cells were determined by Western blot assays. (B) POLD1 guide RNA targeting sequence was presented. The guide RNA targeting region of POLD1 was PCR amplified from genomic DNAs of both wild-type and POLD1-/- cells, and sequence alignment was presented. (C) HepAD38 and HepAD38-POLD1-/- cells were cultured in the presence of 2 mM PFA from day 2 to day 6 after tet removal. From day 6, cccDNA synthesis was initiated by removing PFA for 24 h in the presence or absence of 1 μM APH. Cytoplasmic HBV core DNA and Hirt DNA were extracted and detected by Southern blot hybridization, with mtDNA as a loading control of Hirt DNA. (D) HepAD38 and HepAD38-POLD1-/- cells were cultured in the presence of PFA from day 2 to day 6 after tet removal. At day 4, the cells were re-seeded in 6-well plate and transfected with 2 μg of pcDNA3.1/POLD1-FLAG plasmid, using lipofectamine 3000. From 48 h to 72 h after plasmid transfection (day 6 to day 7 after tet removal), PFA was removed to allow cccDNA synthesis. The cells were harvested at 72 h after transfection, and protein levels of Pol δ1 and β-actin were determined by Western blot assays. Hirt DNA was extracted and HBV DNA was detected by Southern blot analysis, with mtDNA as a loading control. (TIF) [file ppat.1007742.s004.tif]

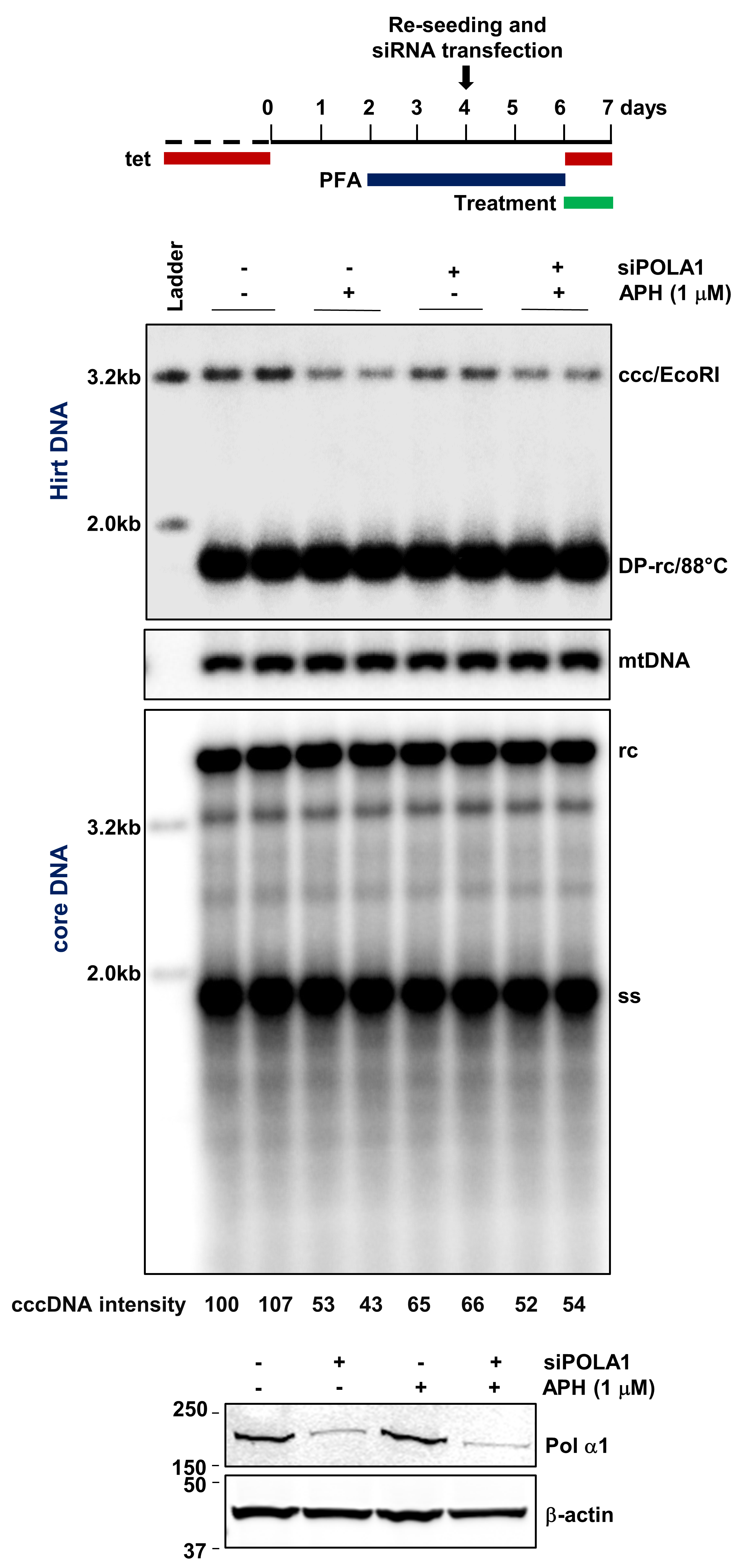

Supplement: S5 Fig — HepAD38 cells were cultured in the presence of 2 mM PFA from day 2 to day 6 after tet removal. At day 4, the cells were re-seeded and transfected with 10 pmol siRNA targeting POLA1 or with scrambled siRNA by using RNAiMAX. From 48 h to 72 h after siRNA transfection (day 6 to day 7 after tet removal), cccDNA synthesis was resumed by removal of PFA from culture medium, followed by DMSO or 1 μM APH treatment. The cells were harvested at 72 h after siRNA transfection, and protein levels of Pol α1 and β-actin were determined by Western blot assays. Cytoplasmic HBV core DNA and Hirt DNA were extracted and detected by Southern blot assays, with mtDNA as a loading control of Hirt DNA. The intensity of HBV cccDNA band was quantified by ImageJ and presented as relative amount in comparison with that in cells transfected with scramble siRNA and treated with DMSO. (TIF) [file ppat.1007742.s005.tif]

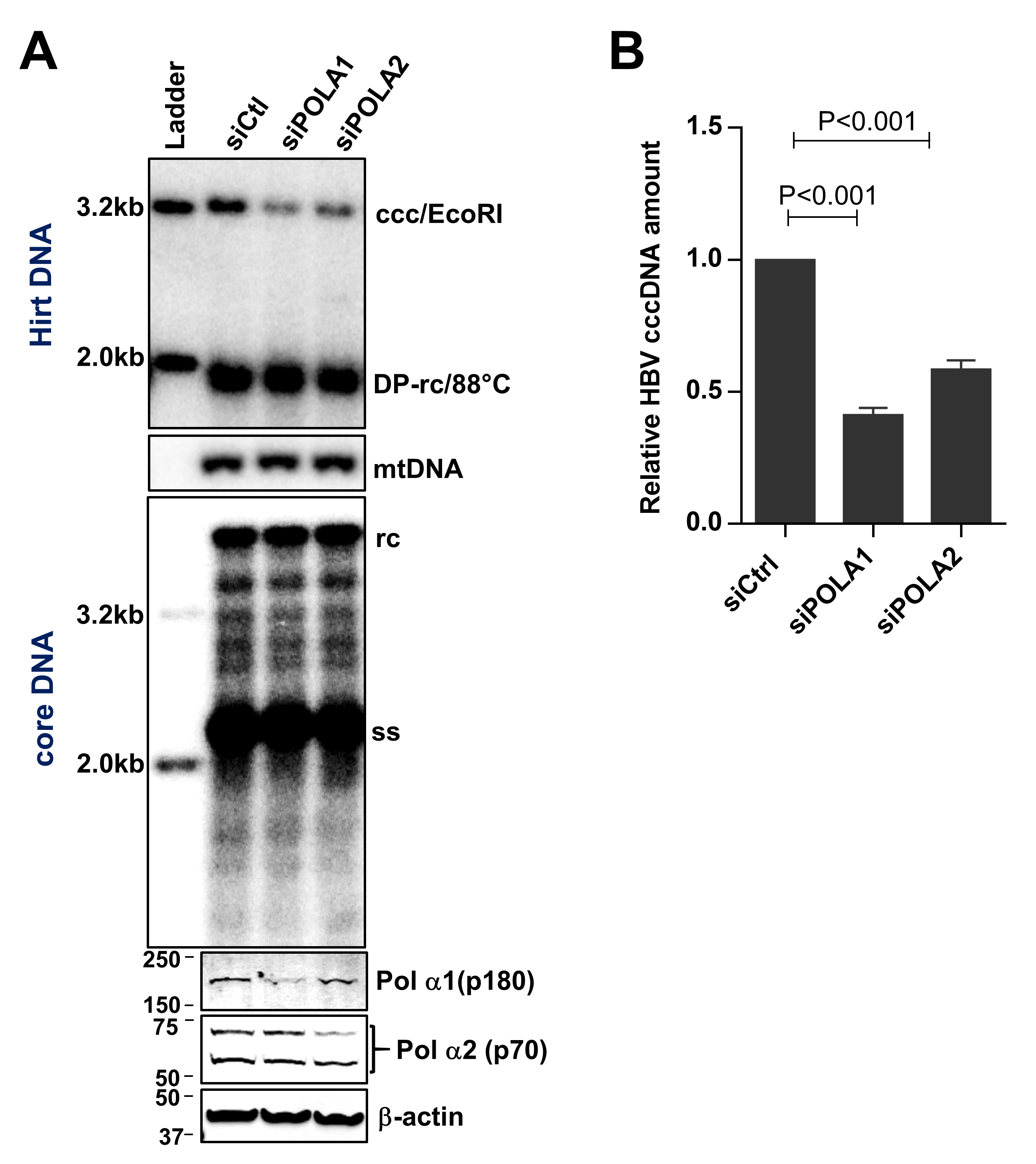

Supplement: S6 Fig — (A) HepAD38 cells were cultured in the presence of PFA from day 2 to day 6 after tet removal. At day 4, the cells were re-seeded and transfected with 10 pmol siRNA targeting POLΑ1, POLA2 or scramble siRNA by using RNAiMAX. From 48 h to 72 h after siRNA transfection (day 6 to day 7 after tet removal), PFA was removed to allow cccDNA synthesis. The cells were harvested at 72 h after siRNA transfection, and protein levels of Pol α1, Pol α2 and β-actin were determined by Western blot assays. Cytoplasmic HBV core DNA and Hirt DNA were extracted and detected by Southern blot analysis, with mtDNA as a loading control of Hirt DNA. (B) The intensity of HBV cccDNA bands was quantified by ImageJ and presented as relative amount in comparison with that in cells transfected with scramble siRNA. Data represent 3 independent experiments. Data were analyzed by two-tailed Student’s t-test (unpaired), P < 0.001. (TIF) [file ppat.1007742.s006.tif]

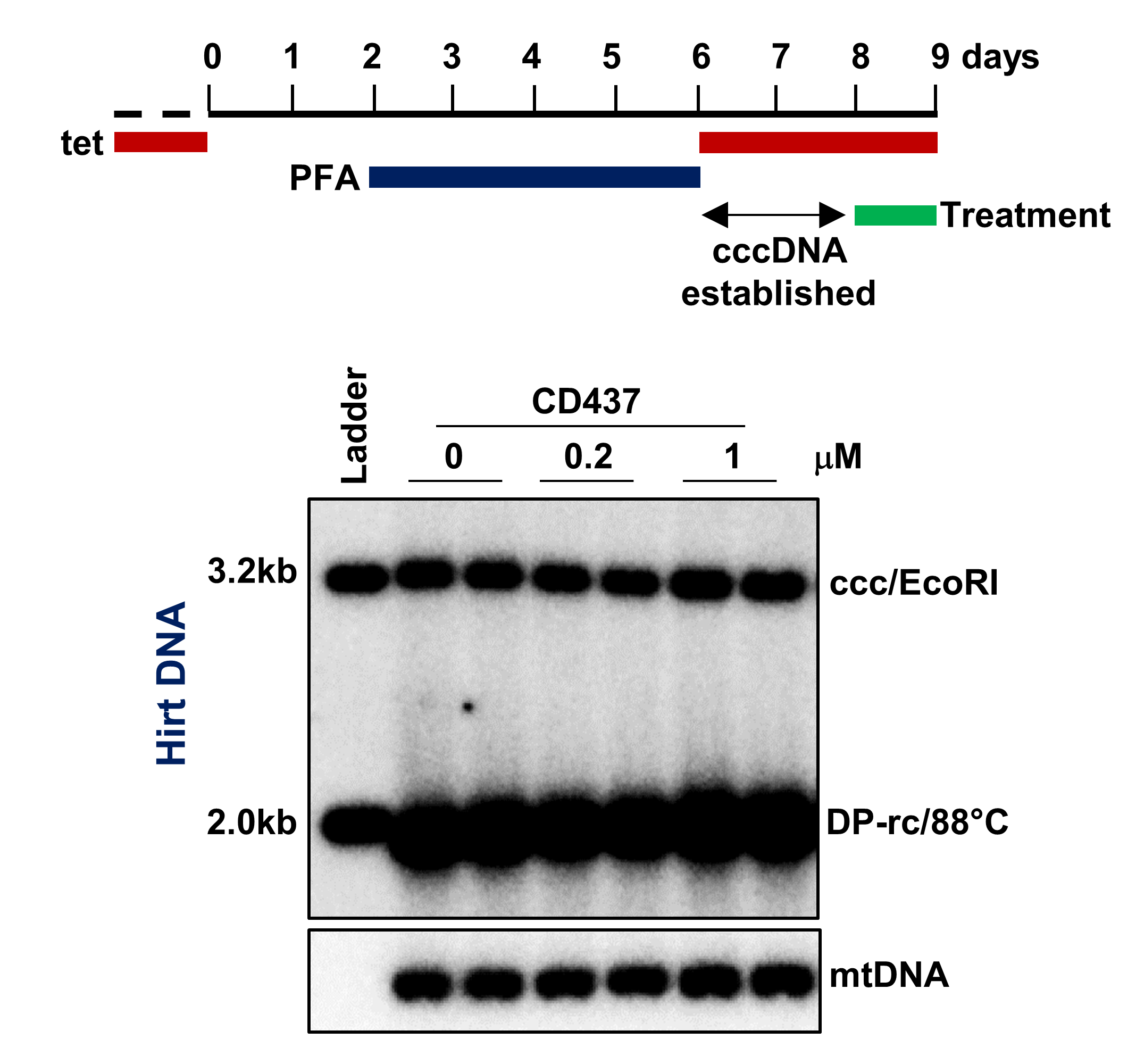

Supplement: S7 Fig — HBV cccDNA pool was allowed to be established for 48 h after removal of PFA and addition of tet on day 6. Cells were then treated with indicated concentrations of CD437 for another 24 h. Hirt DNA was extracted and HBV DNA was detected by Southern blot hybridization, with mtDNA as a loading control. (TIF) [file ppat.1007742.s007.tif]

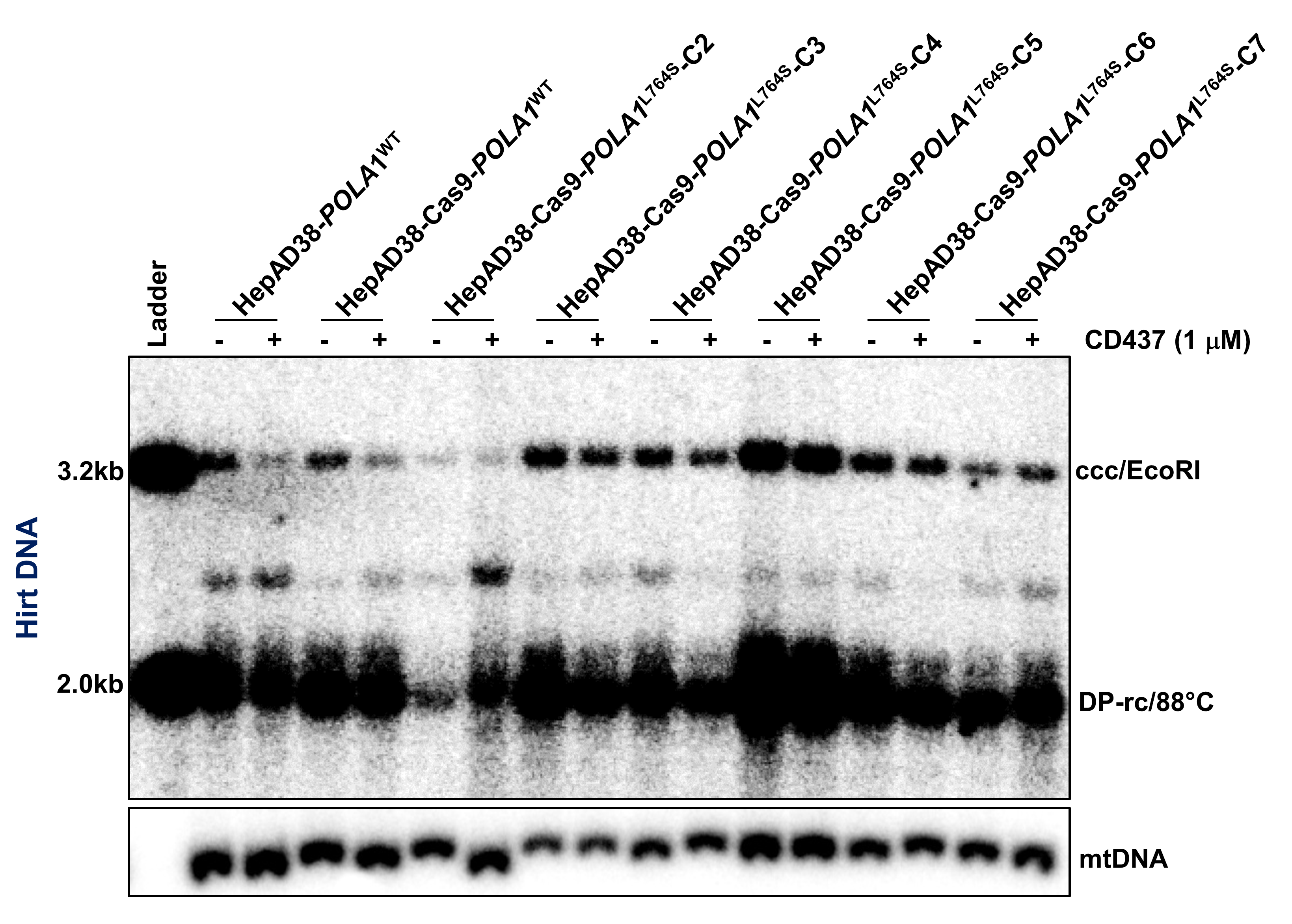

Supplement: S8 Fig — HepAD38, HepAD38-Cas9 and 6 independent clones derived from HepAD38-Cas9 harboring single amino acid mutation of Pol α namely HepAD38-POLΑ1L764S C2 C3 C4 C5 C6 and C7 were cultured in the presence of 2 mM PFA from day 2 to day 6 after tet removal. On day 6, cccDNA synthesis was initiated by removing PFA for 24 h in the presence of 1 μM CD437 treatment. Hirt DNA was extracted and HBV DNA was detected by Southern blot assays with mtDNA as a loading control. (TIF) [file ppat.1007742.s008.tif]

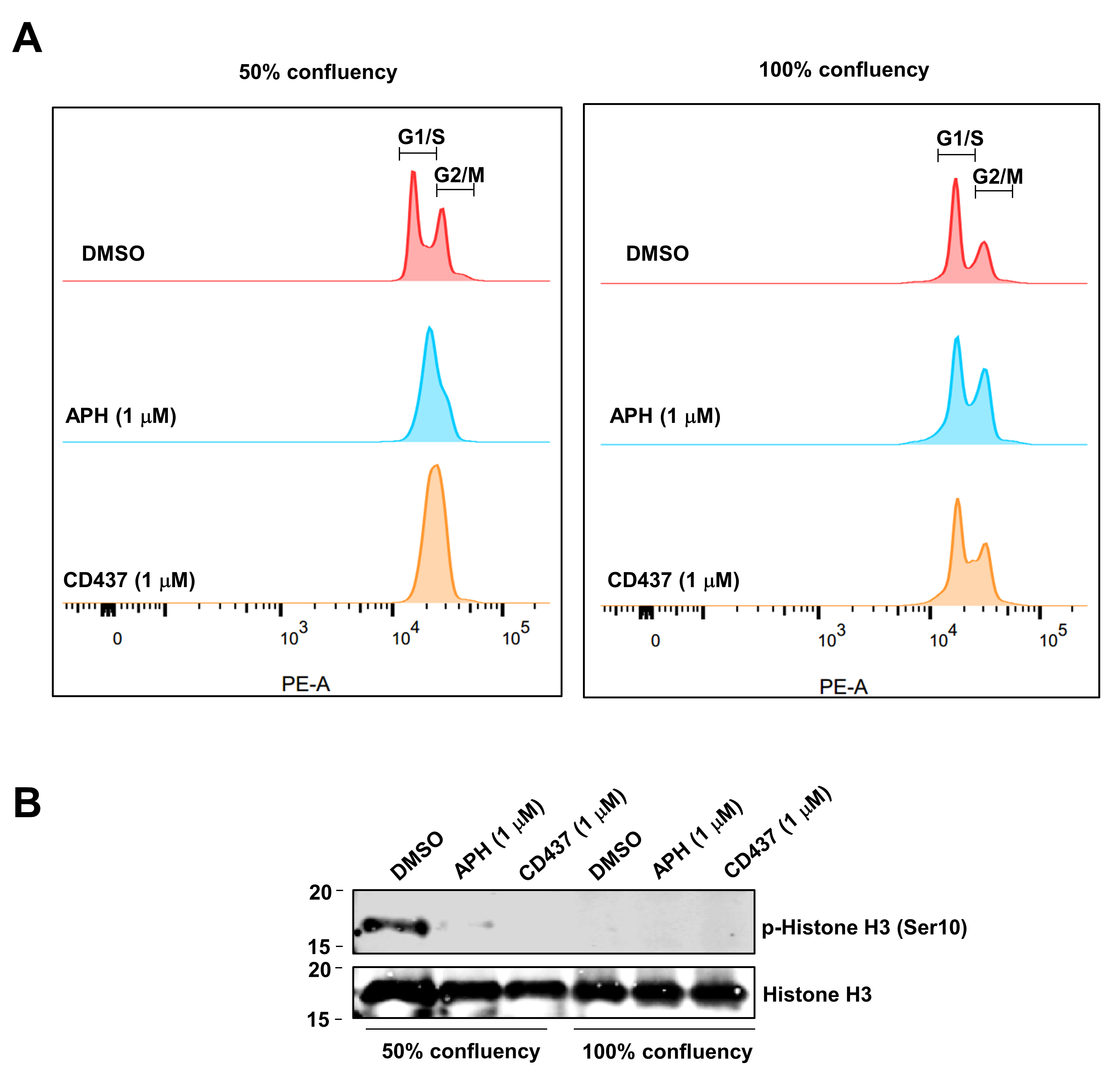

Supplement: S9 Fig — HepAD38 cells at sub-confluent (50%) or confluent (100%) conditions were treated with indicated compounds and harvest after 24 h. (A) Cell cycle stages were determined by flow cytometry with proprium iodide (PI) staining for DNA content. The y-axis indicates cell counts and the x-axis indicates propidium iodide fluorescence intensity. (B) Protein levels of p-Histone H3 (Ser10) and total Histone H3 were determined by Western blot assays. (TIF) [file ppat.1007742.s009.tif]
