# Supplementary material for: DNA Polymerase alpha is essential for intracellular amplification of hepatitis B virus covalently closed circular DNA
Source: PLoS Pathog. 2019 Apr 26;15(4):e1007742. doi: 10.1371/journal.ppat.1007742 (PMC6505960; doi:10.1371/journal.ppat.1007742)
Supplement: S1 Table — (DOCX) [file ppat.1007742.s010.docx]

**S1 Table. Sequence of siRNAs used in the screening of cellular DNA polymerases required for HBV cccDNA amplification.**

| siRNA ID | Target gene | siRNA sequence |
| --- | --- | --- |
| siPOLA-1 | polymerase (DNA directed), alpha | 5'-GCACGCAAUAAAGACAAGA-3’ |
| siPOLB | polymerase (DNA directed), beta | 5'-GCAGCAUCUGUUAUAGCAA-3’ |
| siPOLD1 | polymerase (DNA directed), delta 1, catalytic subunit 125kDa | 5'-CCCUCAAGGUACAAACAUU-3’ |
| siPOLE | polymerase (DNA directed), epsilon | 5'-CGGAAGCAGAUUUAAGGUG-3’ |
| siPOLG | polymerase (DNA directed), gamma | 5'-GGUGCACAGACUUUAUGUA-3’ |
| siPOLH | polymerase (DNA directed), eta | 5'-GCUCGUGCAUUUGGAGUCA-3’ |
| siPOLI | polymerase (DNA directed) iota | 5'-GGAAAUUAUGAUGUGAUGA-3’ |
| siPOLK-1 | polymerase (DNA directed) kappa | 5'-CCAAUAGACAAGCUGUGAU-3’ |
| siPOLL | polymerase (DNA directed), lambda | 5'-GGGAGAAGAAGCAGAAGAG-3’ |
| siPOLM | polymerase (DNA directed), mu | 5'-GCGACACAUGUUGUGAUGG-3’ |
| siPOLN | polymerase (DNA directed) nu | 5'-GCACCCAAUUCAGAUUACU-3’ |
| siPOLQ | polymerase (DNA directed), theta | 5'-GGAAUGCCAUUUUCAAUUA-3’ |
| siPOLZ | polymerase (DNA directed) zeta | 5'-AUGAGUAUGGAUCAUAUAC-3’ |
| siRev1 | Rev1 (DNA directed) | 5'-AUCGGUGGAAUCGGUUUGG-3’ |
