# Supplementary material for: DNA Polymerase alpha is essential for intracellular amplification of hepatitis B virus covalently closed circular DNA
Source: PLoS Pathog. 2019 Apr 26;15(4):e1007742. doi: 10.1371/journal.ppat.1007742 (PMC6505960; doi:10.1371/journal.ppat.1007742)
Supplement: S2 Table — (DOCX) [file ppat.1007742.s011.docx]

**S2 Table. Sequence of primers used in qPCR analysis of cellular and viral RNA and DNA.**

| Gene ID | Primer sequence |
| --- | --- |
| POLA1-sense | 5’-GGACCAACACATCTAGCCTGGA-3’ |
| POLA1-antisense | 5’-GGTCTGGTTTCAAAGCCATTGCC-3’ |
| POLB-sense | 5’-TGCAGAGTCCAGTGGTGACATG-3’ |
| POLB-antisense | 5’-ATGAACCTTTTGTAACTGCTCCAC-3’ |
| POLD1-sense | 5’-ACTACACGGGAGCCACTGTCAT-3’ |
| POLD1-antisense | 5’-GCGTGGTGTAACACAGGTTGTG-3’ |
| POLE-sense | 5’-ACGCTGGAAGAGGTGTATGGCT-3’ |
| POLE-antisense | 5’-GGAACGGTTCTCAGAGATGAGC-3’ |
| POLG-sense | 5’-AGATGGAGAACTTGCGAGCTGC-3’ |
| POLG-antisense | 5’-CACGTCGTTGTAAGGTCCATTGC-3’ |
| POLH-sense | 5’-GGCTGTAGTAAGAACTTCCCAGG-3’ |
| POLH-antisense | 5’-CACGAATGCTCACAACCAGCTG-3’ |
| POLI-sense | 5’-CTACTTCACGCTCTGGCAAGCA-3’ |
| POLI-antisense | 5’-GTGGTATCTAGTGGAGACTCCC-3’ |
| POLK-sense | 5’-CTCCTTGGGTCTAGGTTCAACAC-3’ |
| POLL-sense | 5’-CCATAAGCCTGTCACCTCGTAC-3’ |
| POLL-antisense | 5’-GCTCTCACTGATATGGTCCAGC-3’ |
| POLM-sense | 5’-TGTGAGGAGGTGGAGAGAGTTC-3’ |
| POLM-antisense | 5’-TCGGAGGTCATCTAAGGTTCGC-3’ |
| POLN-sense | 5’-CGAGCAATAACCAGCTTCGAGAG-3’ |
| POLN-antisense | 5’-GGATGAAGGTCTCGCAGAGCAT-3’ |
| POLQ-sense | 5’-CTTGTGGCATCTCCTTGGAGCA-3’ |
| POLQ-antisense | 5’-AATCCCTTGGCTGGTCTCCATC-3’ |
| POLZ-sense | 5’-GTCTGAGACTATTTACCAGGAACC-3’ |
| POLZ-antisense | 5’-CCTTCCAAGGAAAAGTCTCCCTC-3’ |
| Rev1-sense | 5’-GAAATCCACCGAAGAGGAGCAC-3’ |
| Rev1-antisense | 5’-ATCAGGACTGGTCGGCAGATGT-3’ |
| IL-29-sense | 5’-AACTGGGAAGGGCTGCCACATT-3’ |
| IL-29-antisence | 5’-GGAAGACAGGAGAGCTGCAACT-3’ |
| CXCL10-sense | 5’-GGTGAGAAGAGATGTCTGAATCC-3’ |
| CXCL10-anitsense | 5’-GTCCATCCTTGGAAGCACTGCA-3’ |
| TNF-α-sense | 5’-CTCTTCTGCCTGCTGCACTTTG-3’ |
| TNF-α-antisense | 5’-ATGGGCTACAGGCTTGTCACTC-3’ |
| IL-1β-sense | 5’-CCACAGACCTTCCAGGAGAATG-3’ |
| Il-1β-antisense | 5’-GTGCAGTTCAGTGATCGTACAGG-3’ |
| β-actin-sense | 5’-CACCATTGGCAATGAGCGGTTC-3’ |
| β-actin-antisense | 5’-AGGTCTTTGCGGATGTCCACGT-3’ |
| HBV DNA-sense | 5’-TGTACTAGGAGGCTGTAGGC-3’ |
| HBV DNA-antisense | 5’-GGAGACTCTAAGGCTTCCCG-3’ |
| HBV transgene-sense | 5’-AGTAGGCGTGTACGGTGGGAG-3’ |
| HBV transgene-antisense | 5’-TTGGAGGCTTGAACAGTAGG-3’ |
